# Automated Data Harmonization in Clinical Research: Natural Language Processing Approach

**DOI:** 10.2196/75608

**Published:** 2025-08-27

**Authors:** Pratheek Mallya, Ricardo Henao, Chuan Hong, Daniel Wojdyla, Tony Schibler, Vihaan Manchanda, Michael Pencina, Jennifer Hall, Juan Zhao

**Affiliations:** 1 American Heart Association, 7272 Greenville Ave, Dallas, TX, 75231, United States, 1 2147061164; 2 Department of Biostatistics and Bioinformatics, Duke University, Durham, NC, United States; 3 Duke Clinical Research Institute, Durham, NC, United States

**Keywords:** harmonization, natural language processing, cardiovascular research, neural networks, multi-cohort studies

## Abstract

**Background:**

Integrating data is essential for advancing clinical and epidemiological research. However, because datasets often describe variables (eg, demographic and health conditions) in diverse ways, the process of integrating and harmonizing variables from research studies remains a major bottleneck.

**Objective:**

The objective was to assess a natural language processing–based method to automate variable harmonization to achieve a scalable approach to integration of multiple datasets.

**Methods:**

We developed a fully connected neural network (FCN) method, enhanced with contrastive learning, using domain-specific embeddings from the Bidirectional Encoder Representations from Transformers for Biomedical Text Mining language representation model, using 3 cardiovascular datasets: the Atherosclerosis Risk in Communities study, the Framingham Heart Study, and the Multi-Ethnic Study of Atherosclerosis. We used metadata variable descriptions and curated harmonized concepts as ground truth. We framed the problem as a paired sentence classification task. The accuracy of this method was compared with a logistic regression baseline method. To assess the generalizability of the trained models, we also evaluated their performance by separating the 3 datasets when preparing the training and validation sets.

**Results:**

The newly developed FCN achieved a top-5 accuracy of 98.95% (95% CI 98.31%‐99.47%) and an area under the receiver operating characteristic (AUC) of 0.99 (95% CI 0.98‐0.99), outperforming the standard logistic regression model, which exhibited a top-5 accuracy of 22.23% (95% CI 19.91%‐24.87%) and an AUC of 0.82 (95% CI 0.81‐0.83). The contrastive learning enhancement also outperformed the logistic regression model, although slightly below the base FCN model, exhibiting a top-5 accuracy of 89.88% (95% CI 87.88%‐91.68%) and an AUC of 0.98 (95% CI 0.97‐0.98).

**Conclusions:**

This novel approach provides a scalable solution for harmonizing metadata across large-scale cohort studies. The proposed method significantly enhances the performance over the baseline method by using learned representations to categorize harmonized concepts more accurately for cohorts in cardiovascular disease and stroke.

## Introduction

The advent of large language models (LLMs), artificial intelligence, and computational power has the ability to transform our understanding of health and disease. One example is in developing predictive risk models for cardiovascular disease prevention, such as stroke [1]. Machine learning–based stroke risk prediction models enable the inclusion of a wide variety of factors (socioeconomic, behavioral, etc) to assess stroke risk [2]. To fully leverage these approaches and technology, datasets need to be integrated [3,4]. However, integration of datasets is challenging, given inconsistent variable names, column headers, and textual descriptions used to denote clinical or demographic measures [5,6].

These metadata variables, which are the textual labels describing data elements, often differ across studies, even when referring to the same underlying concept (eg, “Systolic_BP” vs “SBP_visit1”). In cardiovascular research, cohort datasets such as the Framingham Heart Study (FHS), the Multi-Ethnic Study of Atherosclerosis (MESA), and the Atherosclerosis Risk in Communities (ARIC) study include thousands of such variables, each with custom naming conventions and sparse documentation. This lack of standardization poses a major challenge for dataset interoperability, phenotyping, and cross-cohort analyses [7].

Data harmonization is the process involving the standardization of disparate variables across multiple datasets into a cohesive and unified format [8,9]. This technique also increases the statistical power of a dataset to solve problems that could not be addressed when using data only from a single study [10]. Traditional harmonization approaches depend heavily on manual mapping by domain experts to map disparate variable descriptions into unified medical concepts, which is time-consuming, error-prone, and difficult to scale [10,11]. Standard vocabularies like Systematized Nomenclature of Medicine–Clinical Terms [12], Logical Observation Identifiers Names and Codes [13], *ICD* (*International Classification of Diseases*) codes [14,15], Current Procedural Terminology [16], Clinical Classifications Software [17], Normalized Names for Clinical Drugs [18], and National Drug Code [19] support structured data harmonization in electronic health records, but are not designed for the free-text, loosely formatted metadata descriptions found in cohort datasets. Recent advances in natural language processing (NLP), including the use of Bidirectional Encoder Representations from Transformers (BERT) models [20,21], knowledge network [22], and other semantic learning methods [23,24], offer promising opportunities to automate the process. Pretrained language models like Bidirectional Encoder Representations from Transformers for Biomedical Text Mining (BioBERT) and semantic embedding techniques can be adapted to understand and categorize medical text [21]. However, these models have not been widely applied to the harmonization of variable-level metadata in observational research settings. Our work addresses this gap.

The goal is to develop and evaluate an NLP-based method for harmonizing variable-level metadata across multiple biomedical datasets. Specifically, we aim to classify free-text variable names and descriptions into harmonized medical concepts that enable integration and analysis across multiple studies.

## Methods

### Overview

The goal of this approach was to combine different datasets by variable definitions into a harmonized variable defined as a medical concept—a term that describes information in a patient’s medical record, such as a diagnosis, a prescription, or a measurement.

To do this, we treated the automation of harmonization as the following steps: (1) to select a list of predefined data harmonization biomedical concepts, and (2) to train a classifier to classify whether a variable belongs to a certain medical concept or not. We used 3 large-scale cardiovascular research cohort studies (ie, FHS, MESA, and ARIC) to harmonize cardiovascular disease risk variables.

For the second step, we used BioBERT embeddings with a fully connected neural network (FCN). BioBERT, a transformer language representation model pretrained on biomedical corpora, generates embeddings for variable descriptions, capturing their semantic relationships [21]. The FCN then classifies these embeddings into predefined harmonized concepts. To address the relatively low number of labeled samples, we also separately augmented the FCN using contrastive learning, a self-supervised representation learning method that is particularly effective in scenarios where training data is limited [25]. The process workflow for this approach is outlined in Multimedia Appendix 1.

### Data Sources

We used the metadata from 3 research cohort datasets—FHS, MESA, and ARIC [7]. The metadata includes variable names and descriptions. In total, we extracted 885 variable descriptions categorized into 64 concepts (spread across 7 concept groups) through manual annotation by 3 independent reviewers, who adapted a preselected list of stroke-related concepts that were illustrated in our previous work [26]. The breakdown of each cohort dataset across cohorts and concept groups is provided in Table 1. The complete list of variable descriptions and their corresponding concepts is detailed in Multimedia Appendix 1. We used this labeled dataset for training and validation.

**Table 1.** Breakdown of the number of variables for each concept group across the 3 study cohorts: Framingham Heart Study, Multi-Ethnic Study of Atherosclerosis, and Atherosclerosis Risk in Communities. The 885 variable descriptions are categorized into 64 concepts across 7 concept groups via manual annotation.

Study	Variables		
	ARIC^a^	MESA^b^	FHS^c^
Total variable descriptions, n	315	161	409
Variables under each category of concept, n (%)			
Sociodemographics	12 (3.8)	11 (6.8)	13 (3.2)
Vitals	18 (5.7)	10 (6.2)	63 (15.4)
Comorbidities	59 (18.7)	76 (47.2)	98 (24)
Laboratories	32 (10.2)	16 (9.9)	49 (12)
Medications	131 (41.6)	30 (18.7)	91 (22.2)
Diet	42 (13.3)	1 (0.6)	74 (18.1)
Other	21 (6.7)	17 (10.6)	21 (5.1)

a ARIC: Atherosclerosis Risk in Communities.

b MESA: Multi-Ethnic Study of Atherosclerosis.

c FHS: Framingham Heart Study.

### BioBERT Embeddings

We used a pretrained BioBERT model to convert the variable descriptions into embedding vectors. BioBERT is a transformer-based model specifically pretrained on large-scale biomedical corpora, including PubMed abstracts and PubMed Central articles [21]. Derived from a general-purpose model known as BERT [20], BioBERT has shown superior performance over BERT for biomedical-related tasks such as Named Entity Recognition [27], Relation Extraction [28], and Question Answering [29]. Particularly, for short-length sequences in the biomedical domain, with pretrained domain knowledge, BioBERT can capture domain-specific semantics and relationships better than a general-purpose model. Given its proven effectiveness in biomedical NLP tasks, BioBERT is an ideal choice for analyzing short-text sequences in the biomedical domain. In this study, we converted each variable description using BioBERT into a 768-dimensional embedding vector for downstream classification.

### Paired Sentences for Classification

We framed the task as a binary classification problem using pairs of variable descriptions (*x_1_*, *x_2_*). Each pair was labeled as either belonging to the same concept or not. We calculated cosine similarity for each pair, and these similarity scores were used to train a supervised classifier to distinguish between matched and nonmatched pairs [30,31].

During inference, for a given variable description, the model compared it against all known concepts. The model calculated similarity scores for each pairing and assigned the description to the concept with the highest similarity score.

### Data Preparation

The dataset was prepared as (1) matching pairs (for every concept, all combinations of variable descriptions belonging to that concept were generated as matched pairs) and (2) nonmatching pairs (for each variable description in a concept, a random sample of descriptions from other concepts was used to generate nonmatching pairs).

To balance the training dataset, we maintained a 1:3 ratio of matching to nonmatching pairs. This ensured sufficient representation of both types of data while maximizing training examples.

### Models

We used the logistic regression model as a baseline classifier. The input was the cosine similarity between BioBERT embedding vectors of paired descriptions [32]. The model was trained using the cross-entropy loss function, and the output was a probabilistic score, which indicates whether the pair represented the same concept (eg, a matched pair or nonmatched pair).

The proposed FCN model consisted of 2 hidden layers, with the first hidden layer having a rectified linear unit activation function [33], and the second layer using a cosine similarity function, rescaled with a weight and a bias parameter, followed by a sigmoid activation function. The framework of the FCN model is outlined in Multimedia Appendix 1. The network was trained using binary cross-entropy loss [34]. The Adam optimizer with early stopping on the validation set was used for optimization [35]. During inference, given a new input variable description, the model calculated similarity scores between the embedding vectors for an input description and each known concept. The concept with the highest score was assigned to the variable description.

### Contrastive Learning

To address the challenges of limited labeled training data, we used a contrastive learning approach [36]. The model was trained to minimize Noise-Contrastive Estimation loss, which improves the representation of variable descriptions by learning from matched and nonmatched pairs [37]. For each variable description, we applied random permutations of embeddings to create augmented pairs. This method further optimized the FCN by leveraging noisy but informative examples. During inference, we used the same methodology as described for the FCN model to categorize an input variable description to a concept.

### Evaluation

To assess the performance, applicability, and generalizability of the method, we used 2 strategies—a combined cohort approach and a separated cohort approach. In the combined cohort approach, we used data from all 3 cohort datasets and randomly split it into training, validation, and testing with an approximate ratio of 4:1:1. For the separated cohort approach, we trained and validated each model on 2 cohorts and used the remaining cohort for testing to assess generalizability across datasets.

We used the area under the receiver operating characteristic (AUC) as our primary performance measure distinguishing matched and nonmatched pairs [38,39]. To evaluate how often the correct concept ranks within the top-K predictions, we used top-1 and top-5 accuracy [40,41]. We used bootstrapping to obtain CIs for both the AUC and accuracy scores [42].

All models were developed using Python (v3.11.5) and PyTorch (v2.2.1). The code and trained models used are found on our GitHub repository: duke-harmonization.

### Ethical Considerations

This study was approved by the Duke University Health System institutional review board (Pro00106364).

For the primary data collections, participants in the original studies provided informed consent, which included provisions for data sharing and secondary use. The datasets used in this study were accessed in accordance with those provisions, and no additional consent was required for this secondary analysis.

All datasets used in this study were fully deidentified and contained no direct or indirect identifiers. The analyses relied exclusively on aggregated metadata, with no linkage to individual-level information. Accordingly, participant confidentiality was maintained throughout.

No participants were directly involved or recruited for this secondary analysis; therefore, no compensation was provided. This paper does not include any images or materials that could lead to the identification of individual participants.

## Results

**Figure 1.**
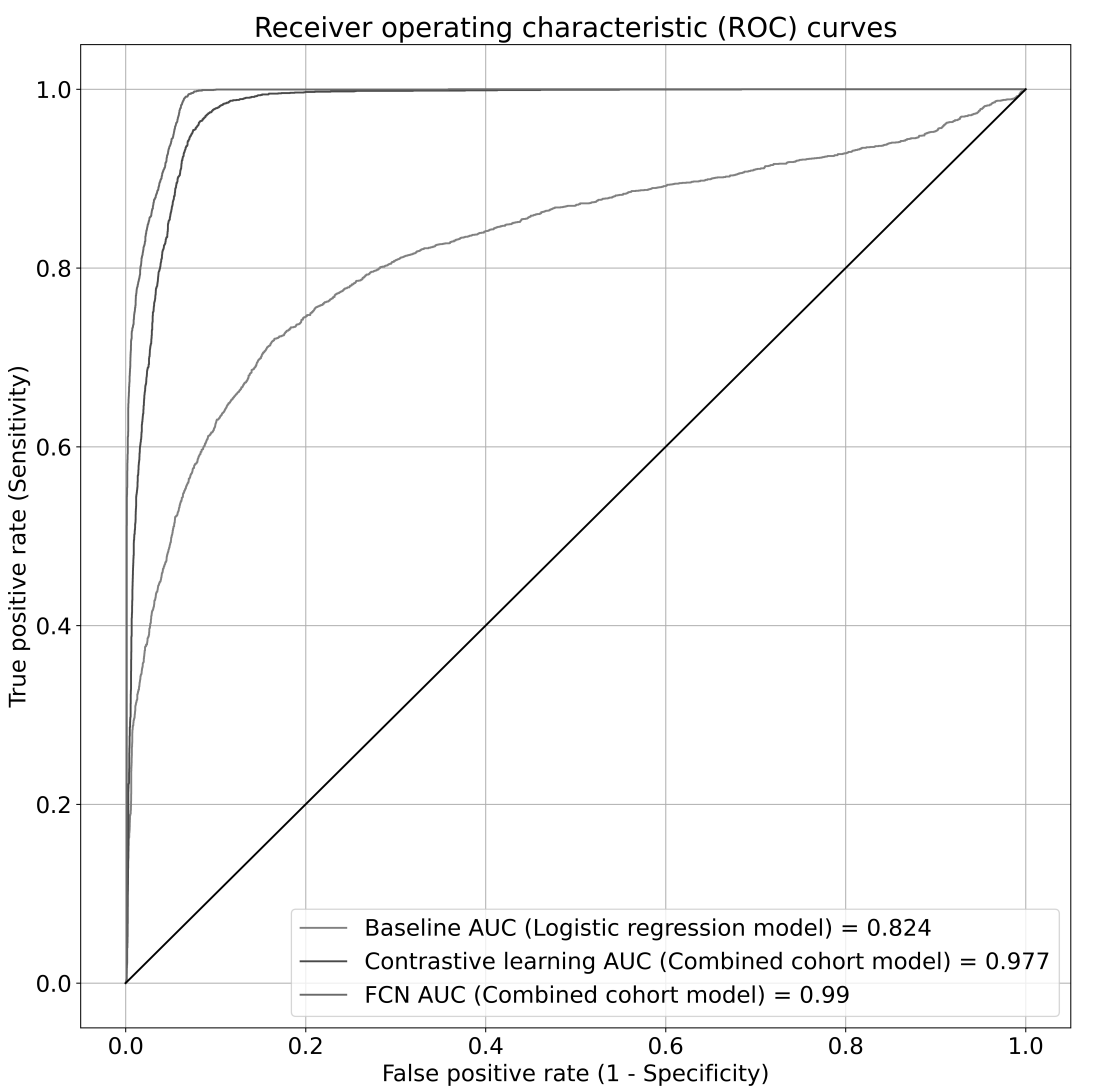
Receiver operating characteristic curves for each of the trained fully connected neural network models and the baseline logistic regression model for the combined cohort approach. In this setting, the variables from all three datasets (Atherosclerosis Risk in Communities, Multi-Ethnic Study of Atherosclerosis, and Framingham Heart Study) were pre-categorized into harmonized concepts. The area under the curve is directly proportional to the model’s performance in distinguishing between matches and nonmatches for a given pair of variable descriptions. The data used to generate the receiver operating characteristic curves consisted of 11,880 pairs of variable descriptions that were absent from the training data, when evaluated on all the cohorts. AUC: area under the receiver operating characteristic; FCN: fully connected neural network.

We extracted a total of 885 variables from 3 datasets, including the FHS, MESA, and ARIC. We precategorized these variables into 64 harmonized concepts and generated 58,890 sentence pairs. In the combined cohort evaluation strategy, we split this dataset into training, validation, and test datasets in a 4:1:1 ratio. The FCN model outperformed the baseline logistic regression model, achieving an AUC of 0.99 (95% CI 0.98‐0.99), compared with the baseline’s AUC of 0.82 (95% CI 0.81‐0.83). The contrastive learning approach achieved an AUC of 0.98 (95% CI 0.97‐0.98), which also outperformed the baseline logistic regression model (Figure 1). For the top-K accuracy, the FCN model achieved a top-1 accuracy of 80.51% (95% CI 78.08%‐83.03%) and a top-5 accuracy of 98.95% (95% CI 98.31%‐99.47%), significantly outperforming the baseline model which achieved top-1 accuracy of 12.12% (95% CI 10.22%‐14.12%) and top-5 accuracy of 22.23% (95% CI 19.91%‐24.87%). The contrastive learning approach achieved a moderate top-1 accuracy score of 63.65% (95% CI 60.59%‐66.81%) and achieved a top-5 accuracy score of 89.88% (95% CI 87.88%‐91.67%; Table 2.

**Table 2.** Top-1 and top-5 accuracy with 95% CIs for baseline logistic regression model, the fully connected neural network model, and the fully connected neural network model with contrastive learning. The evaluation was performed under the combined cohort strategy, where the variables from all 3 cohorts (Atherosclerosis Risk in Communities, Multi-Ethnic Study of Atherosclerosis, and Framingham Heart Study) were precategorized into harmonized concepts.

Model	Top-1 accuracy, % (95% CI)	Top-5 accuracy, % (95% CI)	AUC^a^ (95% CI)
Logistic regression	12.12 (10.22‐14.12)	22.23 (19.91‐24.87)	0.82 (0.81‐0.83)
FCN^b^ (combined cohort)	80.51 (78.08‐83.03)	98.95 (98.31‐99.47)	0.99 (0.98‐0.99)
Contrastive learning	63.65 (60.59‐66.81)	89.88 (87.88‐91.67)	0.98 (0.97‐0.98)

a AUC: area under the receiver operating characteristic.

b FCN: fully connected neural network.

**Figure 2.**
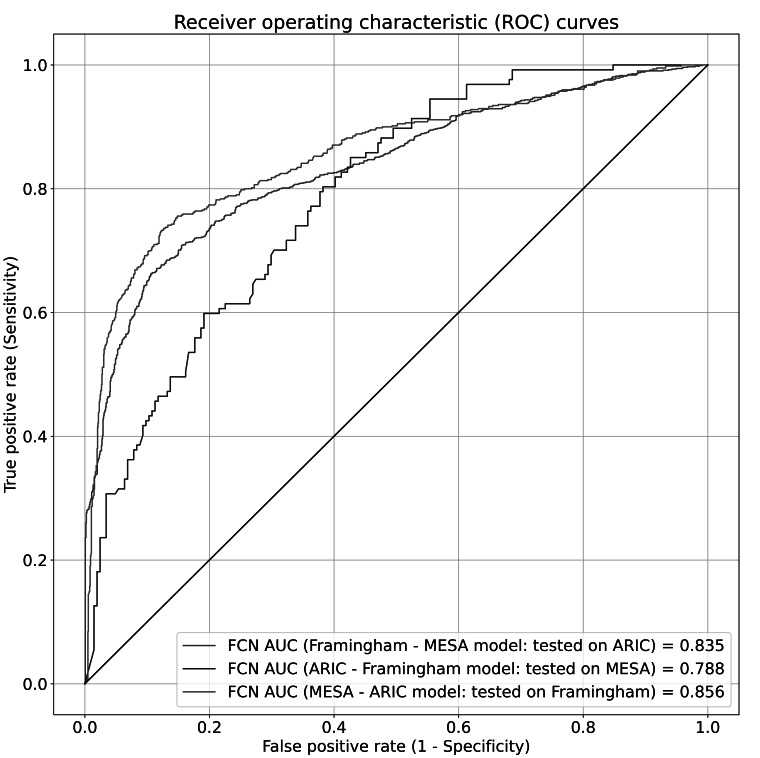
Receiver operating characteristic curves for each of the trained fully connected neural network models for the separated cohort approach. In this setting, the variables from all 3 datasets (Atherosclerosis Risk in Communities, Multi-Ethnic Study of Atherosclerosis, and Framingham Heart Study) were initially precategorized into harmonized concepts. The models were then trained and validated on 2 of the cohorts and then tested on the remaining cohort to assess generalizability of the model across different datasets. The area under the curve is directly proportional to the model’s performance in distinguishing between matches and nonmatches for a given pair of variable descriptions. The receiver operating characteristic curves for each model were obtained by evaluating the model on the subset of the test dataset containing only data from the cohort excluded during training. ARIC: Atherosclerosis Risk in Communities; AUC: area under the receiver operating characteristic; FCN: fully connected neural network; MESA: Multi-Ethnic Study of Atherosclerosis.

We assessed the robustness of FCN in the separated cohort evaluation. The FCN model trained with the ARIC-Framingham model achieved an AUC of 0.78 (95% CI 0.73‐0.83) on the MESA dataset. The MESA-ARIC model, evaluated on the Framingham dataset, achieved the highest AUC of 0.85 (95% CI 0.83‐0.87). The Framingham-MESA model, evaluated on the ARIC dataset, achieved an AUC of 0.83 (95% CI 0.81‐0.85). The ROC curves for the separated cohort models are shown in Figure 2. For the top-K metrics, the ARIC-Framingham model performed best with a top-1 accuracy of 49.33% (95% CI 43.11%‐55.11%) and a top-5 accuracy of 64% (95% CI 57.78%‐69.34%). The MESA-ARIC model performed slightly worse, with a top-1 accuracy of 39.32% (95% CI 35.09%‐43.76%) and a top-5 accuracy of 59.62% (95% CI 55.39%‐64.06%). The Framingham-MESA model exhibited the lowest accuracy performance, with a top-1 accuracy of 32.98% (95% CI 28.23%‐37.47%) and a top-5 accuracy of 48.81% (95% CI 43.79%‐53.56%), which were likely due to greater variability in the ARIC dataset (Table 3).

**Table 3.** Top-1 and top-5 accuracy with 95% CIs for the 3 cohort-specific fully connected neural network models. The evaluation was performed using the separated cohort evaluation strategy, where the variables from all three cohorts (Atherosclerosis Risk in Communities, Multi-Ethnic Study of Atherosclerosis, and Framingham Heart Study) were initially precategorized into harmonized concepts, and the models were then trained and validated on 2 cohorts and tested on the remaining cohort to assess generalizability of the model across different datasets.

Model	Top-1 accuracy, % (95% CI)	Top-5 accuracy, % (95% CI)	AUC^a^ (95% CI)
FCN^b^ (Framingham-MESA^c^), tested on ARIC	32.98 (28.23‐37.47)	48.81 (43.79‐53.56)	0.83 (0.81‐0.85)
FCN (MESA-ARIC^d^) tested on Framingham	39.32 (35.09‐43.76)	59.62 (55.39‐64.06)	0.85 (0.83‐0.87)
FCN (ARIC-Framingham) tested on MESA	49.33 (43.11‐55.11)	64.0 (57.78‐69.34)	0.78 (0.73‐0.83)

a AUC: area under the receiver operating characteristic.

b FCN: fully connected neural network.

c MESA: Multi-Ethnic Study of Atherosclerosis.

d ARIC: Atherosclerosis Risk in Communities.

We plotted the distribution of the predicted score for matches and nonmatches across different concept groups using the baseline method and the FCN model, illustrated in Multimedia Appendix 1. The results indicated that the FCN model generally demonstrates narrower IQRs and more distinct separation between median probabilities for matches and nonmatches, particularly in the diet and sociodemographics categories, which achieved a perfect AUC of 1.0, indicating superior predictive performance compared with the baseline model. The AUC for each model setting when evaluated on a per-concept level is detailed in Multimedia Appendix 1.

We also computed the positive predictive value, negative predictive value, true positive rate, and false positive rate for each of the concepts when using the top-1 predicted concept from the FCN model on the test dataset. The mean positive predictive value across all concepts was 0.78 (SD 0.25), the mean negative predictive value was 0.99 (SD 0.01), the mean true positive rate was 0.85 (SD 0.21), and the mean false positive rate was 0.01 (SD 0.01). The metrics for all concepts are detailed in Multimedia Appendix 1.

## Discussion

### Overview

Harmonizing multiple diverse research cohort datasets can enlarge the data power for training and validating risk prediction models. However, traditional data harmonization techniques need manual comparison, which is time-consuming and barely scalable. This study presents an automated and scalable approach for variable harmonization by leveraging domain-specific NLP and machine learning applied to metadata. We implemented and evaluated the method using the metadata-level variable descriptions from the 3 National Institutes of Health research cohort studies. By reframing variable harmonization as a sentence-pair classification problem, our approach achieves accurate mapping between free-text variable descriptions and standardized concepts, even in the absence of patient-level data. This methodology addresses the common challenges of short text length, sparse annotation, and class imbalance in harmonization tasks.

### Principal Results and Comparison With Previous Work

Our results showed that the FCN model trained on sentence pairs significantly outperformed the baseline logistic regression model. Specifically, both the basic FCN method and the enhanced version using contrastive learning achieved high AUC, top-1, and top-5 accuracy scores, surpassing the logistic regression method. The basic FCN model performed slightly better than the contrastive learning approach. We further assessed the generalizability of our model by separating cohorts for evaluation. Model performance was generally lower and varied, which is expected, due to different variable distributions across different research cohort datasets. The ARIC-Framingham model performed the best in terms of top-K accuracy, suggesting that the MESA dataset shared the most common metadata features with the other two. The Framingham-MESA model performed the worst, possibly because the ARIC metadata has more unique characteristics and models could not effectively learn due to its absence from the training data.

Similar to earlier manual harmonization efforts, our approach began with expert-curated categorization of variables into predefined concepts, which is a foundational step that was essential for the success of the automated classification process, as described in our previous work [26]. However, unlike traditional methods that rely heavily on manual effort throughout, our system automates the subsequent classification, significantly reducing the time and human effort required. While manual harmonization provides expert-driven accuracy, our findings suggest that the automated method can achieve comparable mapping quality with substantially less human input. This framework aligns with practices seen in other harmonization studies, where domain experts played a key role in defining variable concepts [9,43], and other automated harmonization studies [44,45].

Our proposed approach for automated variable harmonization used pretrained embeddings to learn the representations from variable descriptions. Similarly, Yang et al [45] used semantic embeddings and patient-level data to harmonize continuous variables. However, their approach excluded categorical variables and those with missing data. In contrast, our approach uses only variable metadata, thus enabling harmonizing a broader spectrum of variables including both continuous and categorical. Since we use only metadata, this approach also allows harmonization of datasets with or without missing data—thus offering wider applicability for real-world cohort integration.

With the recent advancements in LLMs, Li et al [44] introduced a framework for variable matching using embeddings from general-purpose LLMs. We acknowledge this emerging direction in the field; however, the use of large models often requires fine-tuning on domain-specific data and incurs substantial computational costs, which may limit their practical applicability in resource-constrained settings [46]. By leveraging embeddings from domain-specific LLMs, such as BioBERT, we present a cost-effective approach, requiring fewer computational resources for training and implementation [47].

### Implications for Research and Practice

Our experimental results suggest that our method achieves accurate harmonization for variables across different cardiovascular cohort studies by evaluating contextual similarity across disparate variable descriptions. For example, the descriptions of “diabetes mellitus status” are inconsistent across ARIC, MESA, and Framingham datasets. In the ARIC study, the description varies by visit or exam, such as “diabetes with fasting glucose cutpoint<126” or “diabetes using lower cutpoint 126 mg/dL.” In contrast, Framingham and MESA use descriptions like “diabetes mellitus status, exam 1.” Traditionally, aligning these variables to a SNOMED concept for the condition “diabetes mellitus” requires manual effort and domain expertise, which is difficult to scale across multiple cohorts. Our automated framework significantly reduces this burden, achieving consistent, accurate mapping in a fraction of the time. In practical settings, this approach enables researchers to integrate datasets for cross-cohort analyses, which are essential for predictive modeling and other data-driven applications.

### Limitations

Despite these advancements, we acknowledge that several limitations and challenges remain. First, our proposed framework focused on metadata and did not include patient-level data. However, incorporating patient-level data could help resolve ambiguities in variable definitions. A hybrid approach that leverages patient-level data alongside learned representations from the metadata may help in verifying the automated harmonization results [22]. Another limitation is that we did not address the challenges remaining in the harmonization of different units for laboratory values given that our focus was on metadata and variable descriptions. Incorporating comparisons of variable distributions from patient-level data, in addition to the semantic representations of the variable descriptions, could help alleviate this problem [48]. Future work should explore hybrid methods to combine harmonized variable descriptions with patient-level data to create a more comprehensive and robust framework for cohort integration.

While our study focused on cardiovascular datasets, we acknowledge that the generalizability of the proposed harmonization method to other disease domains or datasets with differing data structures remains unproven. BioBERT is pretrained on large-scale biomedical corpora and thus has potential applicability beyond cardiovascular disease [49], but we recommend validating this approach in other domains, such as oncology, infectious disease, and mental health, where vocabulary, annotation practices, and data sparsity may vary. To improve robustness and portability, we recommend curating preharmonized benchmark datasets for external validation. In addition, future work could explore the integration of lightweight transformers, few-shot learning, or domain-adaptive transfer learning to handle limited labeled data and further extend the applicability of contrastive learning in diverse biomedical settings [50-52].

Third, we did not use more sophisticated models for sequential data such as recurrent neural networks [53], or Long Short-Term Memory networks [54], nor LLMs such as Generative Pre-trained Transformers [55], Pathways Language Model (Google AI) [56], or Large Language Model Meta AI [57], due to the sparse number of labeled examples present in our training data. Application of the contrastive learning approach in tandem with the advanced language models may prove effective in the scalability of the automated harmonization process. Using more complex batch selection methods may also lead to better results via contrastive learning [58].

### Conclusions

In this study, we developed a scalable and automated method for variable harmonization using only metadata from research cohorts. By applying domain-specific language models and framing the task as a sentence-pair classification problem, our approach can accurately map variable descriptions to standardized concepts without needing patient-level data. This reduces the time and effort required for harmonization and is especially useful when access to detailed data is limited. Although we tested the method on cardiovascular datasets, it can potentially be used in other areas like cancer or mental health research. This work provides a foundation for faster and more efficient data integration, which is important for large-scale studies and real-world health research.

## Supplementary material

10.2196/75608 Multimedia Appendix 1 Additional material.

## Abbreviations

ARIC: Atherosclerosis Risk in Communities
AUC: area under the receiver operating characteristic
BERT: Bidirectional Encoder Representations from Transformers
BioBERT: Bidirectional Encoder Representations from Transformers for Biomedical Text Mining
FCN: fully connected neural network
FHS: Framingham Heart Study
*ICD*: *International Classification of Diseases*
LLM: large language model
MESA: Multi-Ethnic Study of Atherosclerosis
NLP: natural language processing
