# Supplementary material for: Automated Data Harmonization in Clinical Research: Natural Language Processing Approach
Source: JMIR Form Res. 2025 Aug 27;9:e75608. doi: 10.2196/75608 (PMC12391522; doi:10.2196/75608)
Supplement: Multimedia Appendix 1 [file formative-v9-e75608-s001.docx]

**
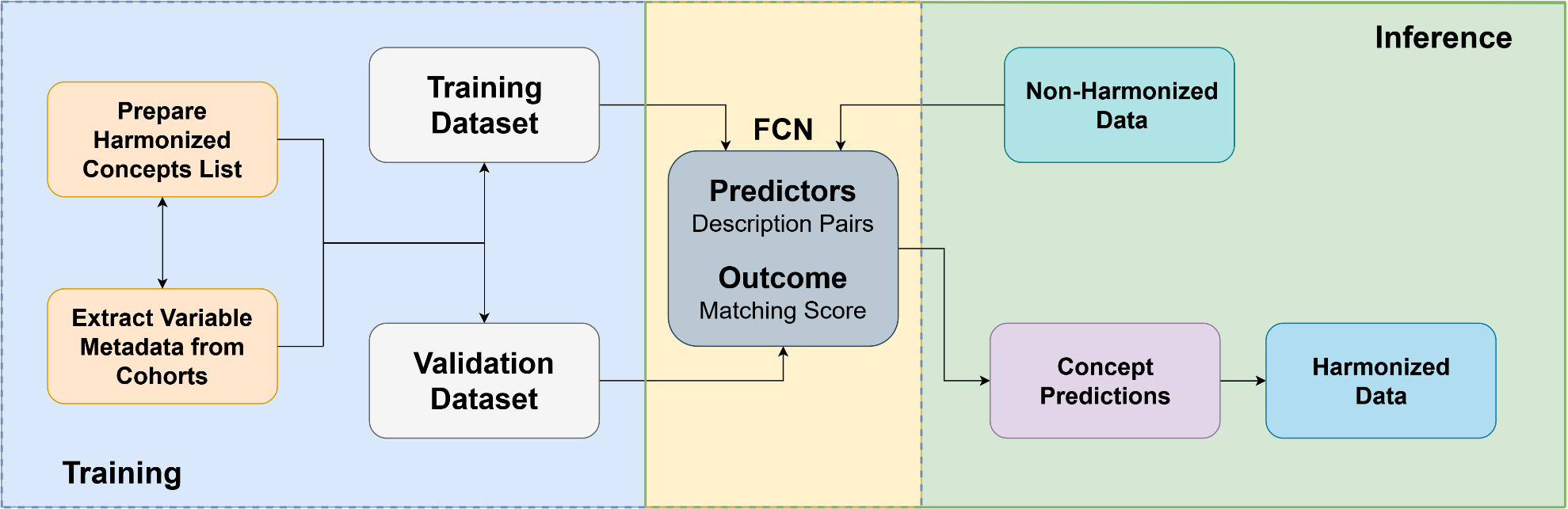
**

**Supplemental Figure 1:** Process workflow for creating an automated harmonization model. From the cohort data, the variable metadata is extracted and categorized into concepts to create the training and validation datasets. These datasets are used to train the model, where the variable description pairs function as the model predictors and the outcome is the prediction score indicating the level of match. The model is then used on the non-harmonized dataset to generate concept predictions for its variables to create the harmonized data during inference.


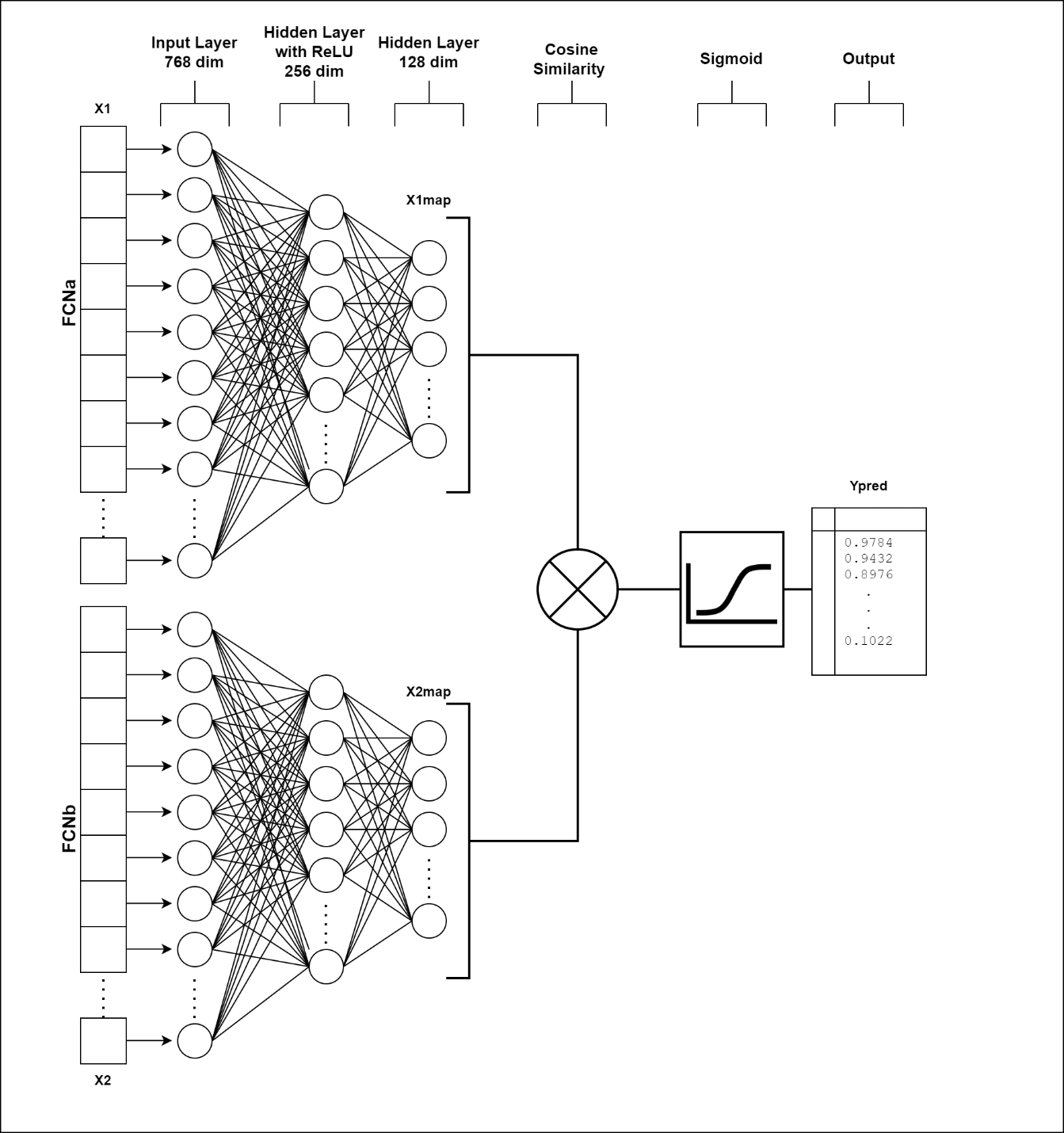


**Supplemental Figure 2:** Model architecture of the neural network. Both networks are identical. The input layer is a 768-dimensional vector BioBERT embedding. The input vectors (*X1*, *X2*) are transformed by the first hidden layer into 256-dimensional vectors followed by a rectified linear unit activation function. These are then transformed by the second hidden layer into 128-dimensional vectors (*X1map*, *X2map*), followed by a cosine similarity operation, which is rescaled using a weight and a bias parameter. This is then transformed by the sigmoid operation to generate the output prediction score (*Ypred*).


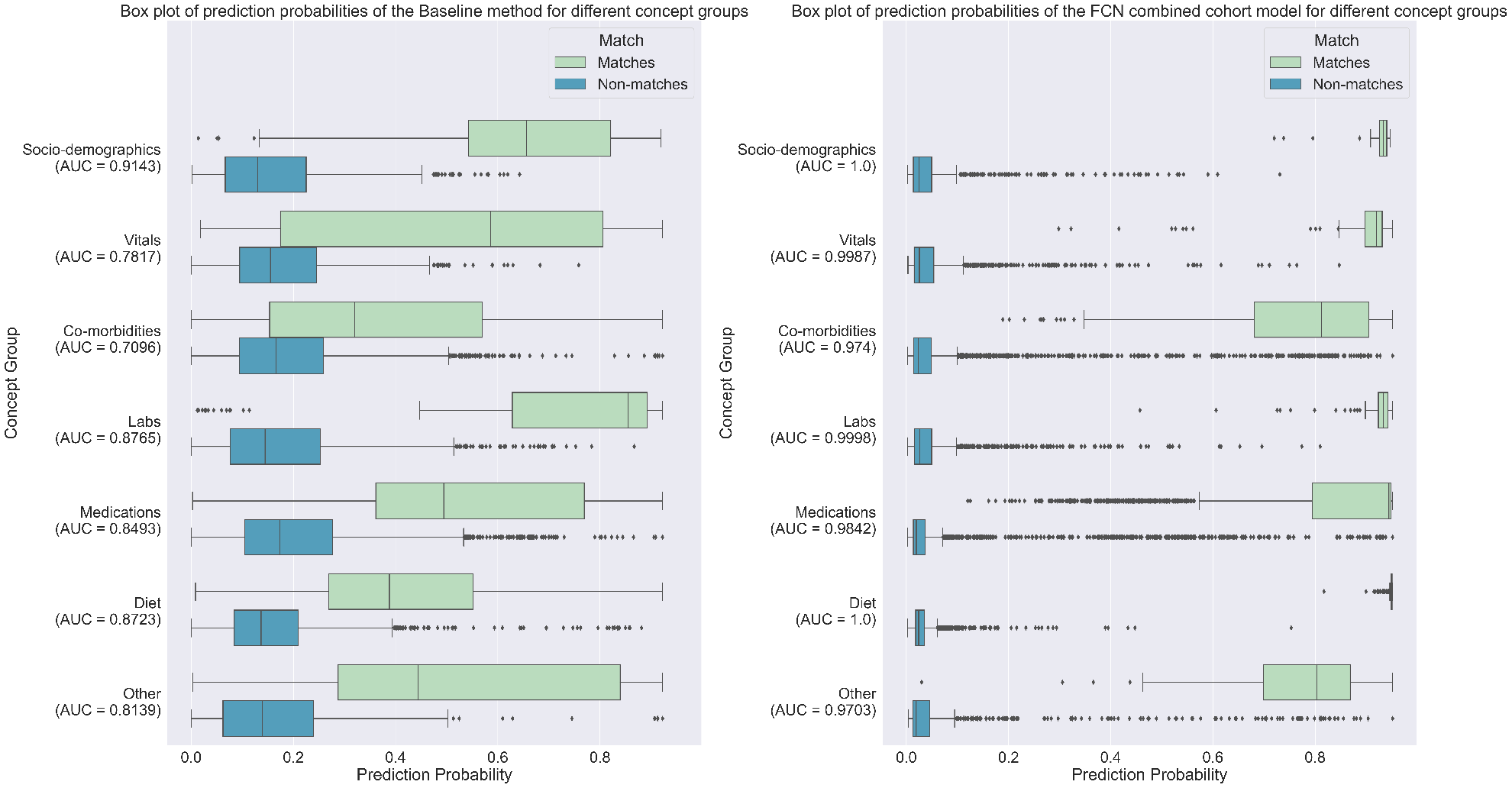


**Supplemental Figure 3:** Box plots of prediction probabilities/scores for the baseline method and the FCN combined cohort method. These contain the distributions of the prediction probabilities for the two methods for all the concepts categorized into groups, as shown in **Table 1**. The combined AUC scores for each concept group are given alongside the group names. The FCN method shows a better discrimination between matches and non-matches across all groups, with an AUC close to one in many categories, showing a clear separation between the inter-quartile ranges for the aforementioned, while the baseline method exhibits a worse performance, specifically for the Vitals and the Co-morbidities concept groups.

**Supplemental Table 1:** Training Dataset containing variable descriptions and their assigned concepts and concept groups.

| **Variable Description** | **Concept Name** | **Concept Description** | **Concept Group** | **Study** |
| --- | --- | --- | --- | --- |
| Atrial fibrillation | AFIB | Atrial fibrillation (0=No, 1=Yes) | Co-morbidities | ARIC |
| Incident AF | AFIB | Atrial fibrillation (0=No, 1=Yes) | Co-morbidities | ARIC |
| time to incident af | AFIB | Atrial fibrillation (0=No, 1=Yes) | Co-morbidities | ARIC |
| DAYS SINCE EXAM 1 | AFIB | Atrial fibrillation (0=No, 1=Yes) | Co-morbidities | ARIC |
| ATRIAL FIBRILLATION | AFIB | Atrial fibrillation (0=No, 1=Yes) | Co-morbidities | Framingham |
| D205-026-ATRIAL-FIBRILLATION | AFIB | Atrial fibrillation (0=No, 1=Yes) | Co-morbidities | Framingham |
| ATRIAL FIBRILLATION ON ECG | AFIB | Atrial fibrillation (0=No, 1=Yes) | Co-morbidities | Framingham |
| ECG-ATRIAL FIBRILLATION | AFIB | Atrial fibrillation (0=No, 1=Yes) | Co-morbidities | Framingham |
| ECG-RHYTHM | AFIB | Atrial fibrillation (0=No, 1=Yes) | Co-morbidities | Framingham |
| ECG - RHYTHM | AFIB | Atrial fibrillation (0=No, 1=Yes) | Co-morbidities | Framingham |
| ATRIAL FIBRILLATION / FLUTTER BY NOVACODE | AFIB | Atrial fibrillation (0=No, 1=Yes) | Co-morbidities | MESA |
| Atrial Fibrillation Diagnosis (via ICD10 Code) | AFIB | Atrial fibrillation (0=No, 1=Yes) | Co-morbidities | MESA |
| Atrial Fibrillation (via ICD10 Code): Time from Baseline to Diagnosis (Days) | AFIB | Atrial fibrillation (0=No, 1=Yes) | Co-morbidities | MESA |
| Time between exam visits, in days | AFIB | Atrial fibrillation (0=No, 1=Yes) | Co-morbidities | MESA |
| Time between first and third visits, in days | AFIB | Atrial fibrillation (0=No, 1=Yes) | Co-morbidities | MESA |
| Time between first and fourth visits, in days | AFIB | Atrial fibrillation (0=No, 1=Yes) | Co-morbidities | MESA |
| ECG ATRIAL FIBRILLATION / FLUTTER | AFIB | Atrial fibrillation (0=No, 1=Yes) | Co-morbidities | MESA |
| TIME BETWEEN FIRST AND FIFTH VISITS, IN DAYS | AFIB | Atrial fibrillation (0=No, 1=Yes) | Co-morbidities | MESA |
| AGE AT VISIT 1 | AGE | Age | Socio-demographics | ARIC |
| Age at Visit 2 | AGE | Age | Socio-demographics | ARIC |
| Age at Visit 3 | AGE | Age | Socio-demographics | ARIC |
| Age at Visit 4 | AGE | Age | Socio-demographics | ARIC |
| Corrected Visit 5 age | AGE | Age | Socio-demographics | ARIC |
| Age (years), Exam 1 | AGE | Age | Socio-demographics | Framingham |
| Age (years), Exam 2 | AGE | Age | Socio-demographics | Framingham |
| Age (years), Exam 3 | AGE | Age | Socio-demographics | Framingham |
| Age (years), Exam 4 | AGE | Age | Socio-demographics | Framingham |
| Age (years), Exam 5 | AGE | Age | Socio-demographics | Framingham |
| Age (years), Exam 6 | AGE | Age | Socio-demographics | Framingham |
| Age (years), Exam 7 | AGE | Age | Socio-demographics | Framingham |
| Age (years), Exam 8 | AGE | Age | Socio-demographics | Framingham |
| Age (years), Exam 9 | AGE | Age | Socio-demographics | Framingham |
| AGE | AGE | Age | Socio-demographics | MESA |
| AGE AT EXAM 2 | AGE | Age | Socio-demographics | MESA |
| AGE AT EXAM3 | AGE | Age | Socio-demographics | MESA |
| Age at exam 4 | AGE | Age | Socio-demographics | MESA |
| AGE AT EXAM 5 | AGE | Age | Socio-demographics | MESA |
| DRINK ALCOHOLIC BEVERAGE Q90 | ALCOHOL | Alcohol (servings per week) | Diet | ARIC |
| NO OF GLASSES WINE CONSUMED P WK Q96 | ALCOHOL | Alcohol (servings per week) | Diet | ARIC |
| NO OF CANS OF BEER CONSUMED P WK Q97 | ALCOHOL | Alcohol (servings per week) | Diet | ARIC |
| NO OF HARD LIQUOR DRINKS PER WK Q98 | ALCOHOL | Alcohol (servings per week) | Diet | ARIC |
| EVER CONSUMED ALCOHOLIC BEVERAGES Q14 | ALCOHOL | Alcohol (servings per week) | Diet | ARIC |
| DRINK ALCOHOL NOW Q15 | ALCOHOL | Alcohol (servings per week) | Diet | ARIC |
| USUAL # OF GLASSES WINE/WK Q17A | ALCOHOL | Alcohol (servings per week) | Diet | ARIC |
| USUAL # OF BEERS PER WEEK Q18A | ALCOHOL | Alcohol (servings per week) | Diet | ARIC |
| USUAL # OF HARD LIQUOR DRINKS/WK Q19A | ALCOHOL | Alcohol (servings per week) | Diet | ARIC |
| Total Alcohol Consumption | ALCOHOL | Alcohol (servings per week) | Diet | Framingham |
| BEER:(servings/week) | ALCOHOL | Alcohol (servings per week) | Diet | Framingham |
| RED WINE:(servings/week) | ALCOHOL | Alcohol (servings per week) | Diet | Framingham |
| WHITE WINE:(servings/week) | ALCOHOL | Alcohol (servings per week) | Diet | Framingham |
| LIQUOR:(servings/week) | ALCOHOL | Alcohol (servings per week) | Diet | Framingham |
| # DRINKS PER WEEK (CURRENT AND FORMER DRINKERS) | ALCOHOL | Alcohol (servings per week) | Diet | MESA |
| Cholesterol lowering medication w/in 2wks: Using 2004 Med Code -V1 | ANYCHOLMED | Taking cholesterol lowering medication (0=No, 1=Yes) | Medications | ARIC |
| Cholesterol lowering medication w/in 2wks: Using 2004 Med Code -V2 | ANYCHOLMED | Taking cholesterol lowering medication (0=No, 1=Yes) | Medications | ARIC |
| Cholesterol lowering medication w/in 2wks: Using 2004 Med Code -V3 | ANYCHOLMED | Taking cholesterol lowering medication (0=No, 1=Yes) | Medications | ARIC |
| Cholesterol lowering medication w/in 2wks: Using 2004 Med Code -V4 | ANYCHOLMED | Taking cholesterol lowering medication (0=No, 1=Yes) | Medications | ARIC |
| Medication taken for high blood cholesterol | ANYCHOLMED | Taking cholesterol lowering medication (0=No, 1=Yes) | Medications | ARIC |
| ANTI-CHOLESTEROL AGENTS | ANYCHOLMED | Taking cholesterol lowering medication (0=No, 1=Yes) | Medications | Framingham |
| D202-016-ANTI-CHOLESTEROL | ANYCHOLMED | Taking cholesterol lowering medication (0=No, 1=Yes) | Medications | Framingham |
| ANTI CHOLESTEROL DRUGS | ANYCHOLMED | Taking cholesterol lowering medication (0=No, 1=Yes) | Medications | Framingham |
| ANTI CHOLESTEROL DRUGS-RESINS | ANYCHOLMED | Taking cholesterol lowering medication (0=No, 1=Yes) | Medications | Framingham |
| ANTI CHOLESTEROL DRUGS-NIACIN | ANYCHOLMED | Taking cholesterol lowering medication (0=No, 1=Yes) | Medications | Framingham |
| ANTI CHOLESTEROL DRUGS-FIBRATES | ANYCHOLMED | Taking cholesterol lowering medication (0=No, 1=Yes) | Medications | Framingham |
| ANTI CHOLESTEROL DRUGS-STATINS | ANYCHOLMED | Taking cholesterol lowering medication (0=No, 1=Yes) | Medications | Framingham |
| ANTI CHOLESTEROL DRUGS-OTHER | ANYCHOLMED | Taking cholesterol lowering medication (0=No, 1=Yes) | Medications | Framingham |
| MEDS - ANTI-CHOL -RESINS | ANYCHOLMED | Taking cholesterol lowering medication (0=No, 1=Yes) | Medications | Framingham |
| MEDS - ANTI-CHOL -NIACIN/NICOTINIC ACID | ANYCHOLMED | Taking cholesterol lowering medication (0=No, 1=Yes) | Medications | Framingham |
| MEDS - ANTI-CHOL -FIBRATES | ANYCHOLMED | Taking cholesterol lowering medication (0=No, 1=Yes) | Medications | Framingham |
| MEDS - ANTI-CHOL -STATINS | ANYCHOLMED | Taking cholesterol lowering medication (0=No, 1=Yes) | Medications | Framingham |
| MEDS - ANTI-CHOL -OTHER | ANYCHOLMED | Taking cholesterol lowering medication (0=No, 1=Yes) | Medications | Framingham |
| MEDS - ANTI-CHOL - RESINS | ANYCHOLMED | Taking cholesterol lowering medication (0=No, 1=Yes) | Medications | Framingham |
| MEDS - ANTI-CHOL - NIACIN/NICOTINIC ACID | ANYCHOLMED | Taking cholesterol lowering medication (0=No, 1=Yes) | Medications | Framingham |
| MEDS - ANTI-CHOL - FIBRATES | ANYCHOLMED | Taking cholesterol lowering medication (0=No, 1=Yes) | Medications | Framingham |
| MEDS - ANTI-CHOL - STATINS | ANYCHOLMED | Taking cholesterol lowering medication (0=No, 1=Yes) | Medications | Framingham |
| MEDS - ANTI-CHOL - OTHER | ANYCHOLMED | Taking cholesterol lowering medication (0=No, 1=Yes) | Medications | Framingham |
| ATC CODE FOR MEDICATION OR FIRST DRUG IN COMPOUND | ANYCHOLMED | Taking cholesterol lowering medication (0=No, 1=Yes) | Medications | Framingham |
| ATC CODE FOR SECOND DRUG IN COMPOUND | ANYCHOLMED | Taking cholesterol lowering medication (0=No, 1=Yes) | Medications | Framingham |
| ATC CODE FOR THIRD DRUG IN COMPOUND | ANYCHOLMED | Taking cholesterol lowering medication (0=No, 1=Yes) | Medications | Framingham |
| ATC CODE FOR FOURTH DRUG IN COMPOUND | ANYCHOLMED | Taking cholesterol lowering medication (0=No, 1=Yes) | Medications | Framingham |
| ATC code for medication or first drug in compound | ANYCHOLMED | Taking cholesterol lowering medication (0=No, 1=Yes) | Medications | Framingham |
| ATC code for medication or second drug in compound | ANYCHOLMED | Taking cholesterol lowering medication (0=No, 1=Yes) | Medications | Framingham |
| ATC code for medication or third drug in compound | ANYCHOLMED | Taking cholesterol lowering medication (0=No, 1=Yes) | Medications | Framingham |
| ATC code for medication or fourth drug in compound | ANYCHOLMED | Taking cholesterol lowering medication (0=No, 1=Yes) | Medications | Framingham |
| HMG CoA reducatace inhibitors (statins) | ANYCHOLMED | Taking cholesterol lowering medication (0=No, 1=Yes) | Medications | MESA |
| Bile-acid sequestrants | ANYCHOLMED | Taking cholesterol lowering medication (0=No, 1=Yes) | Medications | MESA |
| Fibrates | ANYCHOLMED | Taking cholesterol lowering medication (0=No, 1=Yes) | Medications | MESA |
| Miscellaneous lipid-lowering drugs | ANYCHOLMED | Taking cholesterol lowering medication (0=No, 1=Yes) | Medications | MESA |
| Niacin and nictotinic acid | ANYCHOLMED | Taking cholesterol lowering medication (0=No, 1=Yes) | Medications | MESA |
| Probucol | ANYCHOLMED | Taking cholesterol lowering medication (0=No, 1=Yes) | Medications | MESA |
| Any lipid-lowering medication | ANYCHOLMED | Taking cholesterol lowering medication (0=No, 1=Yes) | Medications | MESA |
| HMG COA REDUCATACE INHIBITORS (STATINS) | ANYCHOLMED | Taking cholesterol lowering medication (0=No, 1=Yes) | Medications | MESA |
| BILE-ACID SEQUESTRANTS | ANYCHOLMED | Taking cholesterol lowering medication (0=No, 1=Yes) | Medications | MESA |
| FIBRATES | ANYCHOLMED | Taking cholesterol lowering medication (0=No, 1=Yes) | Medications | MESA |
| MISCELLANEOUS LIPID-LOWERING DRUGS | ANYCHOLMED | Taking cholesterol lowering medication (0=No, 1=Yes) | Medications | MESA |
| NIACIN AND NICTOTINIC ACID | ANYCHOLMED | Taking cholesterol lowering medication (0=No, 1=Yes) | Medications | MESA |
| PROBUCOL | ANYCHOLMED | Taking cholesterol lowering medication (0=No, 1=Yes) | Medications | MESA |
| ANY LIPID-LOWERING MEDICATION | ANYCHOLMED | Taking cholesterol lowering medication (0=No, 1=Yes) | Medications | MESA |
| Used Aspirin-containing analgesics (at Visit 1) in last 2 weeks (0=No, 1=Yes), based on 2004 Med Code | ASPIRIN | Taking aspirin (0=No, 1=Yes) | Medications | ARIC |
| Used Aspirin-containing analgesics (at Visit 2) in last 2 weeks (0=No, 1=Yes), based on 2004 Med Code | ASPIRIN | Taking aspirin (0=No, 1=Yes) | Medications | ARIC |
| Used Aspirin-containing analgesics (at Visit 3) in last 2 weeks (0=No, 1=Yes), based on 2004 Med Code | ASPIRIN | Taking aspirin (0=No, 1=Yes) | Medications | ARIC |
| Used Aspirin-containing analgesics (at Visit 4) in last 2 weeks (0=No, 1=Yes), based on 2004 Med Code | ASPIRIN | Taking aspirin (0=No, 1=Yes) | Medications | ARIC |
| Aspirin taken in last 4 weeks | ASPIRIN | Taking aspirin (0=No, 1=Yes) | Medications | ARIC |
| NUMBER OF ASPIRINS PER TIME INTERVAL | ASPIRIN | Taking aspirin (0=No, 1=Yes) | Medications | Framingham |
| TIME INTERVAL FOR ASPIRIN TAKEN | ASPIRIN | Taking aspirin (0=No, 1=Yes) | Medications | Framingham |
| NUMBER OF ASPIRINS TAKEN REGULARLY | ASPIRIN | Taking aspirin (0=No, 1=Yes) | Medications | Framingham |
| ASPIRIN FREQUENCY | ASPIRIN | Taking aspirin (0=No, 1=Yes) | Medications | Framingham |
| Number aspirins taken regularly | ASPIRIN | Taking aspirin (0=No, 1=Yes) | Medications | Framingham |
| Aspirin Frequency per | ASPIRIN | Taking aspirin (0=No, 1=Yes) | Medications | Framingham |
| ATC CODE FOR MEDICATION OR FIRST DRUG IN COMPOUND | ASPIRIN | Taking aspirin (0=No, 1=Yes) | Medications | Framingham |
| ATC CODE FOR SECOND DRUG IN COMPOUND | ASPIRIN | Taking aspirin (0=No, 1=Yes) | Medications | Framingham |
| ATC CODE FOR THIRD DRUG IN COMPOUND | ASPIRIN | Taking aspirin (0=No, 1=Yes) | Medications | Framingham |
| ATC CODE FOR FOURTH DRUG IN COMPOUND | ASPIRIN | Taking aspirin (0=No, 1=Yes) | Medications | Framingham |
| MEDS - ASPIR, # OF | ASPIRIN | Taking aspirin (0=No, 1=Yes) | Medications | Framingham |
| MEDS - ASPIR, PER DY/WK/MO/YR | ASPIRIN | Taking aspirin (0=No, 1=Yes) | Medications | Framingham |
| ATC code for medication or first drug in compound | ASPIRIN | Taking aspirin (0=No, 1=Yes) | Medications | Framingham |
| ATC code for medication or second drug in compound | ASPIRIN | Taking aspirin (0=No, 1=Yes) | Medications | Framingham |
| ATC code for medication or third drug in compound | ASPIRIN | Taking aspirin (0=No, 1=Yes) | Medications | Framingham |
| ATC code for medication or fourth drug in compound | ASPIRIN | Taking aspirin (0=No, 1=Yes) | Medications | Framingham |
| Aspirin from 280804 (anti-inflam agents) | ASPIRIN | Taking aspirin (0=No, 1=Yes) | Medications | MESA |
| ASPIRIN FROM 280804 (ANTI-INFLAM AGENTS) | ASPIRIN | Taking aspirin (0=No, 1=Yes) | Medications | MESA |
| PREVALENT CORONARY HEART DISEASE | BASE_CVD | Baseline CVD | Other | ARIC |
| SYMPTOMATIC CORONARY HEART DISEASE | BASE_CVD | Baseline CVD | Other | ARIC |
| ROSE INTERMITTENT CLAUDICATION, DEF. 3 | BASE_CVD | Baseline CVD | Other | ARIC |
| Prevalent HF at Visit 1 | BASE_CVD | Baseline CVD | Other | ARIC |
| Cardiovascular Disease (CVD) status | BASE_CVD | Baseline CVD | Other | Framingham |
| Date of Cardiovascular Disease (CVD) status | BASE_CVD | Baseline CVD | Other | Framingham |
| Type of pre-baseline event | BASE_CVD | Baseline CVD | Other | MESA |
| Stroke | BASE_STROKE | Baseline Stroke/TIA (0=No, 1=TIA, 2=Stroke) | Other | ARIC |
| TIA | BASE_STROKE | Baseline Stroke/TIA (0=No, 1=TIA, 2=Stroke) | Other | ARIC |
| Stroke status | BASE_STROKE | Baseline Stroke/TIA (0=No, 1=TIA, 2=Stroke) | Other | Framingham |
| Date of stroke | BASE_STROKE | Baseline Stroke/TIA (0=No, 1=TIA, 2=Stroke) | Other | Framingham |
| Stroke/TIA status | BASE_STROKE | Baseline Stroke/TIA (0=No, 1=TIA, 2=Stroke) | Other | Framingham |
| Date of stroke/TIA | BASE_STROKE | Baseline Stroke/TIA (0=No, 1=TIA, 2=Stroke) | Other | Framingham |
| Blood glucose (mg/dL) (includes fasting and non-fasting), Exam 1 | BG | Blood glucose (mg/dL) (fasting and non-fasting) | Labs | Framingham |
| Blood glucose (mg/dL) (includes fasting and non-fasting), Exam 2 | BG | Blood glucose (mg/dL) (fasting and non-fasting) | Labs | Framingham |
| Blood glucose (mg/dL) (includes fasting and non-fasting), Exam 3 | BG | Blood glucose (mg/dL) (fasting and non-fasting) | Labs | Framingham |
| Blood glucose (mg/dL) (includes fasting and non-fasting), Exam 4 | BG | Blood glucose (mg/dL) (fasting and non-fasting) | Labs | Framingham |
| Blood glucose (mg/dL) (includes fasting and non-fasting), Exam 5 | BG | Blood glucose (mg/dL) (fasting and non-fasting) | Labs | Framingham |
| Blood glucose (mg/dL) (includes fasting and non-fasting), Exam 6 | BG | Blood glucose (mg/dL) (fasting and non-fasting) | Labs | Framingham |
| Blood glucose (mg/dL) (includes fasting and non-fasting), Exam 7 | BG | Blood glucose (mg/dL) (fasting and non-fasting) | Labs | Framingham |
| Blood glucose (mg/dL) (includes fasting and non-fasting), Exam 8 | BG | Blood glucose (mg/dL) (fasting and non-fasting) | Labs | Framingham |
| Blood glucose (mg/dL) (includes fasting and non-fasting), Exam 9 | BG | Blood glucose (mg/dL) (fasting and non-fasting) | Labs | Framingham |
| BODY MASS INDEX IN KG/(M*M) | BMI | Body mass index (kg/m2) | Vitals | ARIC |
| V2 body mass index in kg/m2 | BMI | Body mass index (kg/m2) | Vitals | ARIC |
| Body Mass Index in kg/m**2 | BMI | Body mass index (kg/m2) | Vitals | ARIC |
| Description not found | BMI | Body mass index (kg/m2) | Vitals | ARIC |
| Body mass index (kg/mÃ‚Â²), Exam 1 | BMI | Body mass index (kg/m2) | Vitals | Framingham |
| Body mass index (kg/mÃ‚Â²), Exam 2 | BMI | Body mass index (kg/m2) | Vitals | Framingham |
| Body mass index (kg/mÃ‚Â²), Exam 3 | BMI | Body mass index (kg/m2) | Vitals | Framingham |
| Body mass index (kg/mÃ‚Â²), Exam 4 | BMI | Body mass index (kg/m2) | Vitals | Framingham |
| Body mass index (kg/mÃ‚Â²), Exam 5 | BMI | Body mass index (kg/m2) | Vitals | Framingham |
| Body mass index (kg/mÃ‚Â²), Exam 6 | BMI | Body mass index (kg/m2) | Vitals | Framingham |
| Body mass index (kg/mÃ‚Â²), Exam 7 | BMI | Body mass index (kg/m2) | Vitals | Framingham |
| Body mass index (kg/mÃ‚Â²), Exam 8 | BMI | Body mass index (kg/m2) | Vitals | Framingham |
| Body mass index (kg/mÃ‚Â²), Exam 9 | BMI | Body mass index (kg/m2) | Vitals | Framingham |
| BODY MASS INDEX (kg)/(m^2) | BMI | Body mass index (kg/m2) | Vitals | MESA |
| BODY MASS INDEX (kg/m^2) | BMI | Body mass index (kg/m2) | Vitals | MESA |
| CAROTID BRUITS? Q10 | CARSTEN | Carotid stenosis (0=No, 1=Yes) | Co-morbidities | ARIC |
| Alt. Plaque in any site | CARSTEN | Carotid stenosis (0=No, 1=Yes) | Co-morbidities | ARIC |
| CDI: LEFT CAROTID BRUIT | CARSTEN | Carotid stenosis (0=No, 1=Yes) | Co-morbidities | Framingham |
| CDI: RIGHT CAROTID BRUIT | CARSTEN | Carotid stenosis (0=No, 1=Yes) | Co-morbidities | Framingham |
| CAROTID BRUIT-LEFT | CARSTEN | Carotid stenosis (0=No, 1=Yes) | Co-morbidities | Framingham |
| CAROTID BRUIT-RIGHT | CARSTEN | Carotid stenosis (0=No, 1=Yes) | Co-morbidities | Framingham |
| CAROTID BRUIT - LEFT | CARSTEN | Carotid stenosis (0=No, 1=Yes) | Co-morbidities | Framingham |
| CAROTID BRUIT - RIGHT | CARSTEN | Carotid stenosis (0=No, 1=Yes) | Co-morbidities | Framingham |
| MAXIMUM CAROTID STENOSIS, GRADED | CARSTEN | Carotid stenosis (0=No, 1=Yes) | Co-morbidities | MESA |
| Follow Up Days to Death by 2018 | CENSDAY | Censor day | Other | ARIC |
| Date of death | CENSDAY | Censor day | Other | Framingham |
| Date of last contact | CENSDAY | Censor day | Other | Framingham |
| Time until most recent follow-up (days) | CENSDAY | Censor day | Other | MESA |
| CREATININE (MG-DL) Q9 | CREAT | Creatinine (mg/dL) | Labs | ARIC |
| CREATININE (mg per dL) DEC PT REQ Q8 | CREAT | Creatinine (mg/dL) | Labs | ARIC |
| Creatinine, Serum (mg/dL) | CREAT | Creatinine (mg/dL) | Labs | ARIC |
| Creatinine (mg/dL), Exam 2 | CREAT | Creatinine (mg/dL) | Labs | Framingham |
| Creatinine (mg/dL), Exam 5 | CREAT | Creatinine (mg/dL) | Labs | Framingham |
| Creatinine (mg/dL), Exam 6 | CREAT | Creatinine (mg/dL) | Labs | Framingham |
| Creatinine (mg/dL), Exam 7 | CREAT | Creatinine (mg/dL) | Labs | Framingham |
| Creatinine (mg/dL), Exam 8 | CREAT | Creatinine (mg/dL) | Labs | Framingham |
| Creatinine (mg/dL), Exam 9 | CREAT | Creatinine (mg/dL) | Labs | Framingham |
| CREATININE (mg/dl) | CREAT | Creatinine (mg/dL) | Labs | MESA |
| Creatinine (mg/dl) | CREAT | Creatinine (mg/dL) | Labs | MESA |
| CREATININE | CREAT | Creatinine (mg/dL) | Labs | MESA |
| CURRENT CIGARETTE SMOKER | CURRSMK | Current cigarette smoker (0=No, 1=Yes) | Co-morbidities | ARIC |
| Current Smoker Flag | CURRSMK | Current cigarette smoker (0=No, 1=Yes) | Co-morbidities | ARIC |
| Current Smoker | CURRSMK | Current cigarette smoker (0=No, 1=Yes) | Co-morbidities | ARIC |
| Current Cigarette Smoker | CURRSMK | Current cigarette smoker (0=No, 1=Yes) | Co-morbidities | ARIC |
| V5 Current Cigarette Smoker | CURRSMK | Current cigarette smoker (0=No, 1=Yes) | Co-morbidities | ARIC |
| Current smoking status, Exam 1 | CURRSMK | Current cigarette smoker (0=No, 1=Yes) | Co-morbidities | Framingham |
| Current smoking status, Exam 2 | CURRSMK | Current cigarette smoker (0=No, 1=Yes) | Co-morbidities | Framingham |
| Current smoking status, Exam 3 | CURRSMK | Current cigarette smoker (0=No, 1=Yes) | Co-morbidities | Framingham |
| Current smoking status, Exam 4 | CURRSMK | Current cigarette smoker (0=No, 1=Yes) | Co-morbidities | Framingham |
| Current smoking status, Exam 5 | CURRSMK | Current cigarette smoker (0=No, 1=Yes) | Co-morbidities | Framingham |
| Current smoking status, Exam 6 | CURRSMK | Current cigarette smoker (0=No, 1=Yes) | Co-morbidities | Framingham |
| Current smoking status, Exam 7 | CURRSMK | Current cigarette smoker (0=No, 1=Yes) | Co-morbidities | Framingham |
| Current smoking status, Exam 8 | CURRSMK | Current cigarette smoker (0=No, 1=Yes) | Co-morbidities | Framingham |
| Current smoking status, Exam 9 | CURRSMK | Current cigarette smoker (0=No, 1=Yes) | Co-morbidities | Framingham |
| CIGARETTE SMOKING STATUS | CURRSMK | Current cigarette smoker (0=No, 1=Yes) | Co-morbidities | MESA |
| Cigarrette Smoking Status, Exam 2 | CURRSMK | Current cigarette smoker (0=No, 1=Yes) | Co-morbidities | MESA |
| Cigarrette Smoking Status, Exam 3 | CURRSMK | Current cigarette smoker (0=No, 1=Yes) | Co-morbidities | MESA |
| Cigarette Smoking Status, Exam 4 | CURRSMK | Current cigarette smoker (0=No, 1=Yes) | Co-morbidities | MESA |
| CIGARETTE SMOKING STATUS, EXAM 5 | CURRSMK | Current cigarette smoker (0=No, 1=Yes) | Co-morbidities | MESA |
| DATE OF EVENT | DAYS_SINCE_EXAM1 | Time to event | Other | Framingham |
| Dead by 2018 from DTH/NDI/AFU | DEATH_IND | Death indicator | Other | ARIC |
| Date of death | DEATH_IND | Death indicator | Other | Framingham |
| Death | DEATH_IND | Death indicator | Other | MESA |
| Time to All-Cause Death or End of Year 2015 (days) | DEATH_IND_T2 | Censoring time for all-cause death (only relevant to MESA) | Other | MESA |
| DIABETES w/ fasting glucose cutpt.<126 | DIAB | Diabetes(0=No, 1=Yes) | Co-morbidities | ARIC |
| Diabts22 using chmb07=126 | DIAB | Diabetes(0=No, 1=Yes) | Co-morbidities | ARIC |
| Diabetes Using Lower Cutpoint 126 mg/dL | DIAB | Diabetes(0=No, 1=Yes) | Co-morbidities | ARIC |
| V5 Diabetes - Lower Cutpoint 126 mg/dL | DIAB | Diabetes(0=No, 1=Yes) | Co-morbidities | ARIC |
| Diabetes Mellitus Status, Exam 1 | DIAB | Diabetes(0=No, 1=Yes) | Co-morbidities | Framingham |
| Diabetes Mellitus Status, Exam 2 | DIAB | Diabetes(0=No, 1=Yes) | Co-morbidities | Framingham |
| Diabetes Mellitus Status, Exam 3 | DIAB | Diabetes(0=No, 1=Yes) | Co-morbidities | Framingham |
| Diabetes Mellitus Status, Exam 4 | DIAB | Diabetes(0=No, 1=Yes) | Co-morbidities | Framingham |
| Diabetes Mellitus Status, Exam 5 | DIAB | Diabetes(0=No, 1=Yes) | Co-morbidities | Framingham |
| Diabetes Mellitus Status, Exam 6 | DIAB | Diabetes(0=No, 1=Yes) | Co-morbidities | Framingham |
| Diabetes Mellitus Status, Exam 7 | DIAB | Diabetes(0=No, 1=Yes) | Co-morbidities | Framingham |
| Diabetes Mellitus Status, Exam 8 | DIAB | Diabetes(0=No, 1=Yes) | Co-morbidities | Framingham |
| Diabetes Mellitus Status, Exam 9 | DIAB | Diabetes(0=No, 1=Yes) | Co-morbidities | Framingham |
| Exam 1 Diabetes mellitus by 2003 ADA fasting criteria algorithm | DIAB | Diabetes(0=No, 1=Yes) | Co-morbidities | MESA |
| Exam 2 Diabetes mellitus by 2003 ADA fasting criteria algorithm | DIAB | Diabetes(0=No, 1=Yes) | Co-morbidities | MESA |
| Exam 3 Diabetes mellitus by 2003 ADA fasting criteria algorithm | DIAB | Diabetes(0=No, 1=Yes) | Co-morbidities | MESA |
| Exam 4 Diabetes mellitus by 2003 ADA fasting criteria algorithm | DIAB | Diabetes(0=No, 1=Yes) | Co-morbidities | MESA |
| DIABETES 2003 ADA FASTING CRITERIA | DIAB | Diabetes(0=No, 1=Yes) | Co-morbidities | MESA |
| 2ND AND 3RD DIASTOLIC BP AVERAGE Q22 | DIABP | Seated diastolic blood pressure (MM HG) | Vitals | ARIC |
| 2ND AND 3RD DIASTOLIC BP AVERAGE Q23 | DIABP | Seated diastolic blood pressure (MM HG) | Vitals | ARIC |
| 1ST AND 2ND DIASTOLIC BP AVERAGE Q20 | DIABP | Seated diastolic blood pressure (MM HG) | Vitals | ARIC |
| AVG: Diastolic (mmHg) | DIABP | Seated diastolic blood pressure (MM HG) | Vitals | ARIC |
| SEATED DIASTOLIC BLOOD PRESSURE (mmHg) | DIABP | Seated diastolic blood pressure (MM HG) | Vitals | MESA |
| Seated Diastolic Blood Pressure (mmHg) | DIABP | Seated diastolic blood pressure (MM HG) | Vitals | MESA |
| Seated diastolic blood pressure (mmhg) | DIABP | Seated diastolic blood pressure (MM HG) | Vitals | MESA |
| PHYSICIAN DIASTOLIC BLOOD PRESSURE, 1ST | DIABP1 | PHYSICIAN DIASTOLIC BLOOD PRESSURE, FIRST (MM HG) | Vitals | Framingham |
| D201-050-DIAS-PHYS-FIRST | DIABP1 | PHYSICIAN DIASTOLIC BLOOD PRESSURE, FIRST (MM HG) | Vitals | Framingham |
| FIRST DIASTOLIC BLOOD PRESSURE | DIABP1 | PHYSICIAN DIASTOLIC BLOOD PRESSURE, FIRST (MM HG) | Vitals | Framingham |
| DIASTOLIC BP -PHYSICIAN - 1ST READING | DIABP1 | PHYSICIAN DIASTOLIC BLOOD PRESSURE, FIRST (MM HG) | Vitals | Framingham |
| DIASTOLIC PRESSURE - 1ST MD READING | DIABP1 | PHYSICIAN DIASTOLIC BLOOD PRESSURE, FIRST (MM HG) | Vitals | Framingham |
| PHYSICIAN BP-DIASTOLIC-1ST READING | DIABP1 | PHYSICIAN DIASTOLIC BLOOD PRESSURE, FIRST (MM HG) | Vitals | Framingham |
| PHYSICIAN BP DIASTOLIC - 1ST READING | DIABP1 | PHYSICIAN DIASTOLIC BLOOD PRESSURE, FIRST (MM HG) | Vitals | Framingham |
| PHYSICIAN DIASTOLIC BP - 1ST READING | DIABP1 | PHYSICIAN DIASTOLIC BLOOD PRESSURE, FIRST (MM HG) | Vitals | Framingham |
| MDBP1 - DIASTOLIC 1, MMHG | DIABP1 | PHYSICIAN DIASTOLIC BLOOD PRESSURE, FIRST (MM HG) | Vitals | Framingham |
| PHYSICIAN DIASTOLIC BLOOD PRESSURE, 2ND | DIABP2 | PHYSICIAN DIASTOLIC BLOOD PRESSURE, SECOND (MM HG) | Vitals | Framingham |
| D201-056-DIAS-PHYS-SECOND | DIABP2 | PHYSICIAN DIASTOLIC BLOOD PRESSURE, SECOND (MM HG) | Vitals | Framingham |
| SECOND DIASTOLIC BLOOD PRESSURE | DIABP2 | PHYSICIAN DIASTOLIC BLOOD PRESSURE, SECOND (MM HG) | Vitals | Framingham |
| DIASTOLIC BP -PHYSICIAN - 2ND READING | DIABP2 | PHYSICIAN DIASTOLIC BLOOD PRESSURE, SECOND (MM HG) | Vitals | Framingham |
| DIASTOLIC PRESSURE - 2ND MD READING | DIABP2 | PHYSICIAN DIASTOLIC BLOOD PRESSURE, SECOND (MM HG) | Vitals | Framingham |
| PHYSICIAN BP-2ND READING-DIASTOLIC | DIABP2 | PHYSICIAN DIASTOLIC BLOOD PRESSURE, SECOND (MM HG) | Vitals | Framingham |
| PHYSICIAN BP DIASTOLIC - 2ND READING | DIABP2 | PHYSICIAN DIASTOLIC BLOOD PRESSURE, SECOND (MM HG) | Vitals | Framingham |
| PHYSICIAN DIASTOLIC BP - 2ND READING | DIABP2 | PHYSICIAN DIASTOLIC BLOOD PRESSURE, SECOND (MM HG) | Vitals | Framingham |
| MDBP2 - DIAST, MMHG | DIABP2 | PHYSICIAN DIASTOLIC BLOOD PRESSURE, SECOND (MM HG) | Vitals | Framingham |
| HIGHEST EDUCATION COMPLETED Q54 | EDUCLEV | Education level (1=Less than High School, 2=High School, 3=Some College, 4=College) | Socio-demographics | ARIC |
| D201-099-YEARS-EDUCATION | EDUCLEV | Education level (1=Less than High School, 2=High School, 3=Some College, 4=College) | Socio-demographics | Framingham |
| EDUCATION: HIGHEST LEVEL COMPLETED | EDUCLEV | Education level (1=Less than High School, 2=High School, 3=Some College, 4=College) | Socio-demographics | MESA |
| EVENT NUMBER ON SEQUENCE OF EVENTS | EVENT | Event code | Other | Framingham |
| EVENT NUMBER ON SEQUENCE OF EVENTS | EVENT_DESC | Formatted value of EVENT | Other | Framingham |
| Follow-up HEM Strokes (days) | EVENT_T2_O | Days since exam 1 | Other | ARIC |
| Follow-up CHM Strokes (days) | EVENT_T2_O | Days since exam 1 | Other | ARIC |
| Follow-up ISC Strokes (days) | EVENT_T2_O | Days since exam 1 | Other | ARIC |
| Follow-up DP Strokes (days) | EVENT_T2_O | Days since exam 1 | Other | ARIC |
| Follow-up DPP Strokes (days) | EVENT_T2_O | Days since exam 1 | Other | ARIC |
| Time to Stroke or End of Year 2015 (days) | EVENT_T2_O | Days since exam 1 | Other | MESA |
| Time until most recent follow-up (days) | EVENT_T2_O | Days since exam 1 | Other | MESA |
| Time to All-Cause Death or End of Year 2015 (days) | EVENT_T2_O | Days since exam 1 | Other | MESA |
| Def/Prob Hemmorrhagic Inc. Stroke by Censoring Date | EVENT_VAL_C | Event value (YES/NO) | Other | ARIC |
| Def/Prob Combined Hemm Inc. Stroke by Censoring Date | EVENT_VAL_C | Event value (YES/NO) | Other | ARIC |
| Def/Prob Ischemic Inc. Stroke by Censoring Date | EVENT_VAL_C | Event value (YES/NO) | Other | ARIC |
| Def/Prob Incident Stroke by Censoring Date | EVENT_VAL_C | Event value (YES/NO) | Other | ARIC |
| Def/Prob/Poss Inc. Stroke by Censoring Date | EVENT_VAL_C | Event value (YES/NO) | Other | ARIC |
| Stroke | EVENT_VAL_C | Event value (YES/NO) | Other | MESA |
| Stroke type | EVENT_VAL_C | Event value (YES/NO) | Other | MESA |
| Death | EVENT_VAL_C | Event value (YES/NO) | Other | MESA |
| Death type | EVENT_VAL_C | Event value (YES/NO) | Other | MESA |
| COMBINED FAMILY INCOME GROUP Q62 | FAM_INCOME | Family Income | Socio-demographics | ARIC |
| HOUSEHOLD INCOME LAST YEAR Q6 | FAM_INCOME | Family Income | Socio-demographics | ARIC |
| Current total family income/yr before tx | FAM_INCOME | Family Income | Socio-demographics | Framingham |
| TOTAL GROSS FAMILY INCOME, PAST 12 MONTHS | FAM_INCOME | Family Income | Socio-demographics | MESA |
| FASTING TIME OF 12 HOURS OR MORE | FASTING_12HR | Fasting time of 12 hours or more (0=No, 1=Yes) | Labs | ARIC |
| V2 Fast 12 Hrs or More, Definition 2 | FASTING_12HR | Fasting time of 12 hours or more (0=No, 1=Yes) | Labs | ARIC |
| Fasting Time of 12 Hours or More | FASTING_12HR | Fasting time of 12 hours or more (0=No, 1=Yes) | Labs | ARIC |
| FASTING TIME OF 8 HOURS OR MORE | FASTING_8HR | Fasting time of 8 hours or more (0=No, 1=Yes) | Labs | ARIC |
| V2 Fast 8 Hrs or More, Definition 2 | FASTING_8HR | Fasting time of 8 hours or more (0=No, 1=Yes) | Labs | ARIC |
| Fasting Time of 8 Hours or More | FASTING_8HR | Fasting time of 8 hours or more (0=No, 1=Yes) | Labs | ARIC |
| Fasting (>= 8 hours) blood glucose (mg/dL), Exam 3 | FASTING_BG | Fasting blood glucose (>: 8 hours) (mg/dL) | Labs | Framingham |
| Fasting (>= 8 hours) blood glucose (mg/dL), Exam 4 | FASTING_BG | Fasting blood glucose (>: 8 hours) (mg/dL) | Labs | Framingham |
| Fasting (>= 8 hours) blood glucose (mg/dL), Exam 5 | FASTING_BG | Fasting blood glucose (>: 8 hours) (mg/dL) | Labs | Framingham |
| Fasting (>= 8 hours) blood glucose (mg/dL), Exam 6 | FASTING_BG | Fasting blood glucose (>: 8 hours) (mg/dL) | Labs | Framingham |
| Fasting (>= 8 hours) blood glucose (mg/dL), Exam 7 | FASTING_BG | Fasting blood glucose (>: 8 hours) (mg/dL) | Labs | Framingham |
| Fasting (>= 8 hours) blood glucose (mg/dL), Exam 8 | FASTING_BG | Fasting blood glucose (>: 8 hours) (mg/dL) | Labs | Framingham |
| Fasting (>= 8 hours) blood glucose (mg/dL), Exam 9 | FASTING_BG | Fasting blood glucose (>: 8 hours) (mg/dL) | Labs | Framingham |
| Exam 1 Fasting Glucose- Calibrated | FASTING_BG | Fasting blood glucose (>: 8 hours) (mg/dL) | Labs | MESA |
| FASTING GLUCOSE (mg/dL) CALIBRATED | FASTING_BG | Fasting blood glucose (>: 8 hours) (mg/dL) | Labs | MESA |
| Fasting Glucose (mg/dL) Calibrated | FASTING_BG | Fasting blood glucose (>: 8 hours) (mg/dL) | Labs | MESA |
| FASTING GLUCOSE (mg/dl) | FASTING_BG | Fasting blood glucose (>: 8 hours) (mg/dL) | Labs | MESA |
| New Maternal History of Stroke | FH_STROKE | Family history of stroke, Mother or Father (0=No, 1=Yes) | Co-morbidities | ARIC |
| New Paternal History of Stroke | FH_STROKE | Family history of stroke, Mother or Father (0=No, 1=Yes) | Co-morbidities | ARIC |
| FATHER-HAVE STROKE BEFORE AGE 65 | FH_STROKE | Family history of stroke, Mother or Father (0=No, 1=Yes) | Co-morbidities | Framingham |
| MOTHER-HAVE STROKE BEFORE AGE 65 | FH_STROKE | Family history of stroke, Mother or Father (0=No, 1=Yes) | Co-morbidities | Framingham |
| FAMILY HISTORY OF STROKE: PARENT | FH_STROKE | Family history of stroke, Mother or Father (0=No, 1=Yes) | Co-morbidities | MESA |
| CONSUMED APPLES OR PEARS Q9 | FRUITS | Fruits (servings per week) | Diet | ARIC |
| CONSUMED ORANGES Q10 | FRUITS | Fruits (servings per week) | Diet | ARIC |
| CONSUMED PEACHES APRICOTS PLUMS Q12 | FRUITS | Fruits (servings per week) | Diet | ARIC |
| CONSUMED BANANAS Q13 | FRUITS | Fruits (servings per week) | Diet | ARIC |
| CONSUMED OTHER FRUITS Q14 | FRUITS | Fruits (servings per week) | Diet | ARIC |
| CONSUMED APPLES OR PEARS Q9 | FRUITS | Fruits (servings per week) | Diet | ARIC |
| CONSUMED ORANGES Q10 | FRUITS | Fruits (servings per week) | Diet | ARIC |
| CONSUMED PEACHES APRICOTS PLUMS Q12 | FRUITS | Fruits (servings per week) | Diet | ARIC |
| CONSUMED BANANAS Q13 | FRUITS | Fruits (servings per week) | Diet | ARIC |
| CONSUMED OTHER FRUITS Q14 | FRUITS | Fruits (servings per week) | Diet | ARIC |
| FRESH APPLE OR PAIR | FRUITS | Fruits (servings per week) | Diet | Framingham |
| ORANGE OR TANGERINE | FRUITS | Fruits (servings per week) | Diet | Framingham |
| HALF GRAPEFRUIT OR GLASS JUICE | FRUITS | Fruits (servings per week) | Diet | Framingham |
| PEACHES,APRICOTS,PLUMS OR NECTARINES | FRUITS | Fruits (servings per week) | Diet | Framingham |
| BANANAS | FRUITS | Fruits (servings per week) | Diet | Framingham |
| FRESH,FROZEN OR CANNED STRAWBERRIES | FRUITS | Fruits (servings per week) | Diet | Framingham |
| BLACKBERRIES,BLUEBERRIES,RASPBERRIES | FRUITS | Fruits (servings per week) | Diet | Framingham |
| CANTELOUPE OR HONEYDEW MELON-SLICE | FRUITS | Fruits (servings per week) | Diet | Framingham |
| SLICE OF WATERMELON | FRUITS | Fruits (servings per week) | Diet | Framingham |
| FRESH OR CANNED PINEAPPLE | FRUITS | Fruits (servings per week) | Diet | Framingham |
| FRESH OR CANNED CHERRIES | FRUITS | Fruits (servings per week) | Diet | Framingham |
| HALF CUP PAPAYAS | FRUITS | Fruits (servings per week) | Diet | Framingham |
| ONE QUARTER OF AN AVOCADO | FRUITS | Fruits (servings per week) | Diet | Framingham |
| HALF CUP RAISINS OR DRY APRICOTS | FRUITS | Fruits (servings per week) | Diet | Framingham |
| HALF CUP DATES OR FIGS | FRUITS | Fruits (servings per week) | Diet | Framingham |
| SMALL PACK RAISINS OR GRAPES | FRUITS | Fruits (servings per week) | Diet | Framingham |
| RAISINS:(servings/week) | FRUITS | Fruits (servings per week) | Diet | Framingham |
| PRUNES:(servings/week) | FRUITS | Fruits (servings per week) | Diet | Framingham |
| BANANAS:(servings/week) | FRUITS | Fruits (servings per week) | Diet | Framingham |
| CANTALOUPE:(servings/week) | FRUITS | Fruits (servings per week) | Diet | Framingham |
| WATERMELON:(servings/week) | FRUITS | Fruits (servings per week) | Diet | Framingham |
| APPLES/PEARS, FRESH:(servings/week) | FRUITS | Fruits (servings per week) | Diet | Framingham |
| ORANGES:(servings/week) | FRUITS | Fruits (servings per week) | Diet | Framingham |
| GRAPEFRUIT:(servings/week) | FRUITS | Fruits (servings per week) | Diet | Framingham |
| STRAWBERRIES:(servings/week) | FRUITS | Fruits (servings per week) | Diet | Framingham |
| BLUEBERRIES:(servings/week) | FRUITS | Fruits (servings per week) | Diet | Framingham |
| PEACHES:(servings/week) | FRUITS | Fruits (servings per week) | Diet | Framingham |
| HEALTH CONDITION Q9 | GENHLTH | General Health (visit 1) | Co-morbidities | ARIC |
| YOUR HEALTH NOW IN GENERAL | GENHLTH | General Health (visit 1) | Co-morbidities | Framingham |
| Rate general health | GENHLTH2 | General Health (visit 5) | Co-morbidities | ARIC |
| IN GENERAL, HOW IS YOUR HEALTH | GENHLTH2 | General Health (visit 5) | Co-morbidities | Framingham |
| SF-12 - HOW YOUR HLTH | GENHLTH2 | General Health (visit 5) | Co-morbidities | Framingham |
| IN GENERAL YOUR HEALTH IS | GENHLTH2 | General Health (visit 5) | Co-morbidities | MESA |
| DERIVED GLUCOSE VALUE in mg/dl | GLUCOSE | Glucose value in mg/dL | Labs | ARIC |
| GLUCOSE (mg per dL) DEC PT REQ Q7 | GLUCOSE | Glucose value in mg/dL | Labs | ARIC |
| GLUCOSE (VALUE IN MG/DL) Q4A | GLUCOSE | Glucose value in mg/dL | Labs | ARIC |
| FASTING GLUCOSE (VALUE) Q4A | GLUCOSE | Glucose value in mg/dL | Labs | ARIC |
| Fasting Glucose (mg/dL) | GLUCOSE | Glucose value in mg/dL | Labs | ARIC |
| RE-CALIBRATED HDL CHOL. in mg/dl | HDL | HDL cholesterol | Labs | ARIC |
| HDL CHOLESTREROL (VALUE) Q3A | HDL | HDL cholesterol | Labs | ARIC |
| HDL CHOLESTEROL (VALUE IN MG/DL) Q3A | HDL | HDL cholesterol | Labs | ARIC |
| HDL CHOLESTEROL (VALUE) Q3A | HDL | HDL cholesterol | Labs | ARIC |
| HDLC-Cholesterol (mg/dL) | HDL | HDL cholesterol | Labs | ARIC |
| HDL cholesterol (mg/dL), Exam 1 | HDL | HDL cholesterol | Labs | Framingham |
| HDL cholesterol (mg/dL), Exam 2 | HDL | HDL cholesterol | Labs | Framingham |
| HDL cholesterol (mg/dL), Exam 3 | HDL | HDL cholesterol | Labs | Framingham |
| HDL cholesterol (mg/dL), Exam 4 | HDL | HDL cholesterol | Labs | Framingham |
| HDL cholesterol (mg/dL), Exam 5 | HDL | HDL cholesterol | Labs | Framingham |
| HDL cholesterol (mg/dL), Exam 6 | HDL | HDL cholesterol | Labs | Framingham |
| HDL cholesterol (mg/dL), Exam 7 | HDL | HDL cholesterol | Labs | Framingham |
| HDL cholesterol (mg/dL), Exam 8 | HDL | HDL cholesterol | Labs | Framingham |
| HDL cholesterol (mg/dL), Exam 9 | HDL | HDL cholesterol | Labs | Framingham |
| HDL CHOLESTROL (mg/dl) | HDL | HDL cholesterol | Labs | MESA |
| HDL CHOLESTEROL (mg/dl) | HDL | HDL cholesterol | Labs | MESA |
| HDL Cholesterol (mg/dl) | HDL | HDL cholesterol | Labs | MESA |
| STANDING HEIGHT TO NEAREST CM Q1 | HGT_CM | Standing height (to the nearest cm) | Vitals | ARIC |
| Description not found | HGT_CM | Standing height (to the nearest cm) | Vitals | ARIC |
| Standing height (cm) | HGT_CM | Standing height (to the nearest cm) | Vitals | ARIC |
| Height (inches), Exam 1 | HGT_CM | Standing height (to the nearest cm) | Vitals | Framingham |
| Height (inches), Exam 2 | HGT_CM | Standing height (to the nearest cm) | Vitals | Framingham |
| Height (inches), Exam 3 | HGT_CM | Standing height (to the nearest cm) | Vitals | Framingham |
| Height (inches), Exam 4 | HGT_CM | Standing height (to the nearest cm) | Vitals | Framingham |
| Height (inches), Exam 5 | HGT_CM | Standing height (to the nearest cm) | Vitals | Framingham |
| Height (inches), Exam 6 | HGT_CM | Standing height (to the nearest cm) | Vitals | Framingham |
| Height (inches), Exam 7 | HGT_CM | Standing height (to the nearest cm) | Vitals | Framingham |
| Height (inches), Exam 8 | HGT_CM | Standing height (to the nearest cm) | Vitals | Framingham |
| Height (inches), Exam 9 | HGT_CM | Standing height (to the nearest cm) | Vitals | Framingham |
| HEIGHT (cm) | HGT_CM | Standing height (to the nearest cm) | Vitals | MESA |
| HYPERTENSION LOWERING MED. USE, DEF. 1 | HRX | Taking blood pressure lowering medication (0=No, 1=Yes) | Co-morbidities | ARIC |
| Hypertension Meds (Self reported) | HRX | Taking blood pressure lowering medication (0=No, 1=Yes) | Co-morbidities | ARIC |
| V3 HYPERTENSION MEDICATIONS, DEF. 1 | HRX | Taking blood pressure lowering medication (0=No, 1=Yes) | Co-morbidities | ARIC |
| V4 Hypert Med in Past 2 Wks: Self-rptd | HRX | Taking blood pressure lowering medication (0=No, 1=Yes) | Co-morbidities | ARIC |
| Medication taken for high blodd pressure or hypertension | HRX | Taking blood pressure lowering medication (0=No, 1=Yes) | Co-morbidities | ARIC |
| Treated for hypertension, Exam 1 | HRX | Taking blood pressure lowering medication (0=No, 1=Yes) | Co-morbidities | Framingham |
| Treated for hypertension, Exam 2 | HRX | Taking blood pressure lowering medication (0=No, 1=Yes) | Co-morbidities | Framingham |
| Treated for hypertension, Exam 3 | HRX | Taking blood pressure lowering medication (0=No, 1=Yes) | Co-morbidities | Framingham |
| Treated for hypertension, Exam 4 | HRX | Taking blood pressure lowering medication (0=No, 1=Yes) | Co-morbidities | Framingham |
| Treated for hypertension, Exam 5 | HRX | Taking blood pressure lowering medication (0=No, 1=Yes) | Co-morbidities | Framingham |
| Treated for hypertension, Exam 6 | HRX | Taking blood pressure lowering medication (0=No, 1=Yes) | Co-morbidities | Framingham |
| Treated for hypertension, Exam 7 | HRX | Taking blood pressure lowering medication (0=No, 1=Yes) | Co-morbidities | Framingham |
| Treated for hypertension, Exam 8 | HRX | Taking blood pressure lowering medication (0=No, 1=Yes) | Co-morbidities | Framingham |
| Treated for hypertension, Exam 9 | HRX | Taking blood pressure lowering medication (0=No, 1=Yes) | Co-morbidities | Framingham |
| Hypertension Medication | HRX | Taking blood pressure lowering medication (0=No, 1=Yes) | Co-morbidities | MESA |
| Any hypertension medication | HRX | Taking blood pressure lowering medication (0=No, 1=Yes) | Co-morbidities | MESA |
| ANY HYPERTENSION MEDICATION | HRX | Taking blood pressure lowering medication (0=No, 1=Yes) | Co-morbidities | MESA |
| ROSE INTERMITTENT CLAUDICATION, DEF. 3 | HXCVD | History of cardiovascular disease | Co-morbidities | ARIC |
| PREVALENT CORONARY HEART DISEASE | HXCVD | History of cardiovascular disease | Co-morbidities | ARIC |
| ROSE ANGINA | HXCVD | History of cardiovascular disease | Co-morbidities | ARIC |
| Prevalent HF at Visit 1 | HXCVD | History of cardiovascular disease | Co-morbidities | ARIC |
| Follow-up time for INCHF18 in days | HXCVD | History of cardiovascular disease | Co-morbidities | ARIC |
| Incident HF (from discharge codes) | HXCVD | History of cardiovascular disease | Co-morbidities | ARIC |
| Prevalent CHD at V3, definition 3 | HXCVD | History of cardiovascular disease | Co-morbidities | ARIC |
| DAYS SINCE EXAM 1 | HXCVD | History of cardiovascular disease | Co-morbidities | ARIC |
| REPORT ROSE ANGINA IN AFU? Q1 | HXCVD | History of cardiovascular disease | Co-morbidities | ARIC |
| Prevalent CHD at V4, definition 3 | HXCVD | History of cardiovascular disease | Co-morbidities | ARIC |
| Cardiovascular Disease (CVD) status | HXCVD | History of cardiovascular disease | Co-morbidities | Framingham |
| Date of Cardiovascular Disease (CVD) status | HXCVD | History of cardiovascular disease | Co-morbidities | Framingham |
| DATE OF EVENT | HXCVD | History of cardiovascular disease | Co-morbidities | Framingham |
| EVENT NUMBER ON SEQUENCE OF EVENTS | HXCVD | History of cardiovascular disease | Co-morbidities | Framingham |
| Date of Exam 2 | HXCVD | History of cardiovascular disease | Co-morbidities | Framingham |
| Date of Exam 3 | HXCVD | History of cardiovascular disease | Co-morbidities | Framingham |
| Date of Exam 4 | HXCVD | History of cardiovascular disease | Co-morbidities | Framingham |
| Date of Exam 5 | HXCVD | History of cardiovascular disease | Co-morbidities | Framingham |
| Date of Exam 6 | HXCVD | History of cardiovascular disease | Co-morbidities | Framingham |
| Date of Exam 7 | HXCVD | History of cardiovascular disease | Co-morbidities | Framingham |
| Date of Exam 8 | HXCVD | History of cardiovascular disease | Co-morbidities | Framingham |
| Date of Exam 9 | HXCVD | History of cardiovascular disease | Co-morbidities | Framingham |
| Myocardial Infarction (MI) | HXCVD | History of cardiovascular disease | Co-morbidities | MESA |
| Time to MI or End of Year 2015 (days) | HXCVD | History of cardiovascular disease | Co-morbidities | MESA |
| Resuscitated Cardiac Arrest | HXCVD | History of cardiovascular disease | Co-morbidities | MESA |
| Time to RCA or End of Year 2015 (days) | HXCVD | History of cardiovascular disease | Co-morbidities | MESA |
| Angina Pectoris | HXCVD | History of cardiovascular disease | Co-morbidities | MESA |
| Time to Angina or End of Year 2015 (days) | HXCVD | History of cardiovascular disease | Co-morbidities | MESA |
| Congestive Heart Failure (CHF) | HXCVD | History of cardiovascular disease | Co-morbidities | MESA |
| Time to CHF or End of Year 2015 (days) | HXCVD | History of cardiovascular disease | Co-morbidities | MESA |
| Peripheral Vascular Disease (PVD) | HXCVD | History of cardiovascular disease | Co-morbidities | MESA |
| Time to PVD or End of Year 2015 (days) | HXCVD | History of cardiovascular disease | Co-morbidities | MESA |
| Type of pre-baseline event | HXCVD | History of cardiovascular disease | Co-morbidities | MESA |
| WHO ROSE Intermittent Claudication | HXCVD | History of cardiovascular disease | Co-morbidities | MESA |
| Time between exam visits, in days | HXCVD | History of cardiovascular disease | Co-morbidities | MESA |
| Any hypertension medication | HXCVD | History of cardiovascular disease | Co-morbidities | MESA |
| Time between first and third visits, in days | HXCVD | History of cardiovascular disease | Co-morbidities | MESA |
| Time between first and fourth visits, in days | HXCVD | History of cardiovascular disease | Co-morbidities | MESA |
| TIME BETWEEN FIRST AND FIFTH VISITS, IN DAYS | HXCVD | History of cardiovascular disease | Co-morbidities | MESA |
| PAD V1, Def 2 (same for both genders) | HXHRTD | History of heart disease | Co-morbidities | ARIC |
| ROSE ANGINA | HXHRTD | History of heart disease | Co-morbidities | ARIC |
| Prevalent HF at Visit 1 | HXHRTD | History of heart disease | Co-morbidities | ARIC |
| Follow-up time for INCHF18 in days | HXHRTD | History of heart disease | Co-morbidities | ARIC |
| Incident HF (from discharge codes) | HXHRTD | History of heart disease | Co-morbidities | ARIC |
| Follow up time for PROC18 (days) | HXHRTD | History of heart disease | Co-morbidities | ARIC |
| Cardiac Procedures | HXHRTD | History of heart disease | Co-morbidities | ARIC |
| PAD V3, Def 2 (same for both genders) | HXHRTD | History of heart disease | Co-morbidities | ARIC |
| DAYS SINCE EXAM 1 | HXHRTD | History of heart disease | Co-morbidities | ARIC |
| REPORT ROSE ANGINA IN AFU? Q1 | HXHRTD | History of heart disease | Co-morbidities | ARIC |
| PAD V4, Def 2 (same for both genders) | HXHRTD | History of heart disease | Co-morbidities | ARIC |
| Cardiovascular Disease (CVD) status | HXHRTD | History of heart disease | Co-morbidities | Framingham |
| Date of Cardiovascular Disease (CVD) status | HXHRTD | History of heart disease | Co-morbidities | Framingham |
| DATE OF EVENT | HXHRTD | History of heart disease | Co-morbidities | Framingham |
| EVENT NUMBER ON SEQUENCE OF EVENTS | HXHRTD | History of heart disease | Co-morbidities | Framingham |
| Date of cardiovascular procedure | HXHRTD | History of heart disease | Co-morbidities | Framingham |
| PROCEDURE NUMBER | HXHRTD | History of heart disease | Co-morbidities | Framingham |
| Date of Exam 2 | HXHRTD | History of heart disease | Co-morbidities | Framingham |
| Date of Exam 3 | HXHRTD | History of heart disease | Co-morbidities | Framingham |
| Date of Exam 4 | HXHRTD | History of heart disease | Co-morbidities | Framingham |
| Date of Exam 5 | HXHRTD | History of heart disease | Co-morbidities | Framingham |
| Date of Exam 6 | HXHRTD | History of heart disease | Co-morbidities | Framingham |
| Date of Exam 7 | HXHRTD | History of heart disease | Co-morbidities | Framingham |
| Date of Exam 8 | HXHRTD | History of heart disease | Co-morbidities | Framingham |
| Date of Exam 9 | HXHRTD | History of heart disease | Co-morbidities | Framingham |
| Myocardial Infarction (MI) | HXHRTD | History of heart disease | Co-morbidities | MESA |
| Time to MI or End of Year 2015 (days) | HXHRTD | History of heart disease | Co-morbidities | MESA |
| Resuscitated Cardiac Arrest | HXHRTD | History of heart disease | Co-morbidities | MESA |
| Time to RCA or End of Year 2015 (days) | HXHRTD | History of heart disease | Co-morbidities | MESA |
| Angina Pectoris | HXHRTD | History of heart disease | Co-morbidities | MESA |
| Time to Angina or End of Year 2015 (days) | HXHRTD | History of heart disease | Co-morbidities | MESA |
| Congestive Heart Failure (CHF) | HXHRTD | History of heart disease | Co-morbidities | MESA |
| Time to CHF or End of Year 2015 (days) | HXHRTD | History of heart disease | Co-morbidities | MESA |
| Peripheral Vascular Disease (PVD) | HXHRTD | History of heart disease | Co-morbidities | MESA |
| Time to PVD or End of Year 2015 (days) | HXHRTD | History of heart disease | Co-morbidities | MESA |
| Coronary Bypass Graft (CBG) | HXHRTD | History of heart disease | Co-morbidities | MESA |
| Time to CBG or End of Year 2015 (days) | HXHRTD | History of heart disease | Co-morbidities | MESA |
| PTCA, Cor. Stent, or Cor. Atherectomy | HXHRTD | History of heart disease | Co-morbidities | MESA |
| Time to PTCA or End of Year 2015 (days) | HXHRTD | History of heart disease | Co-morbidities | MESA |
| Coronary Revascularization | HXHRTD | History of heart disease | Co-morbidities | MESA |
| Time to Cor. Revasc. or End of Year 2015 (days) | HXHRTD | History of heart disease | Co-morbidities | MESA |
| Type of pre-baseline event | HXHRTD | History of heart disease | Co-morbidities | MESA |
| Time between exam visits, in days | HXHRTD | History of heart disease | Co-morbidities | MESA |
| Time between first and third visits, in days | HXHRTD | History of heart disease | Co-morbidities | MESA |
| Time between first and fourth visits, in days | HXHRTD | History of heart disease | Co-morbidities | MESA |
| TIME BETWEEN FIRST AND FIFTH VISITS, IN DAYS | HXHRTD | History of heart disease | Co-morbidities | MESA |
| MD DIAGNOSED MYOCARDIAL INFARCTION | HXMI | History of MI(REGARDS model) | Co-morbidities | ARIC |
| HISTORY OF MYOCARDIAL INFARCTION | HXMI | History of MI(REGARDS model) | Co-morbidities | ARIC |
| Follow up time for MI18 (days) | HXMI | History of MI(REGARDS model) | Co-morbidities | ARIC |
| Follow up time for INC_BY18 (days) | HXMI | History of MI(REGARDS model) | Co-morbidities | ARIC |
| MI by Censoring Date | HXMI | History of MI(REGARDS model) | Co-morbidities | ARIC |
| MI/FATCHD by Censoring Date | HXMI | History of MI(REGARDS model) | Co-morbidities | ARIC |
| V2 MD Diagnosed Myocardial Infarction | HXMI | History of MI(REGARDS model) | Co-morbidities | ARIC |
| V2 History of Myocardial Infarction | HXMI | History of MI(REGARDS model) | Co-morbidities | ARIC |
| DAYS SINCE EXAM 1 | HXMI | History of MI(REGARDS model) | Co-morbidities | ARIC |
| V3 MD Diagnosed Myocardial Infarction | HXMI | History of MI(REGARDS model) | Co-morbidities | ARIC |
| V3 History of Myocardial Infarction | HXMI | History of MI(REGARDS model) | Co-morbidities | ARIC |
| V4 MD Diagnosed Myocardial Infarction | HXMI | History of MI(REGARDS model) | Co-morbidities | ARIC |
| V4 History of Myocardial Infarction | HXMI | History of MI(REGARDS model) | Co-morbidities | ARIC |
| DATE OF EVENT | HXMI | History of MI(REGARDS model) | Co-morbidities | Framingham |
| EVENT NUMBER ON SEQUENCE OF EVENTS | HXMI | History of MI(REGARDS model) | Co-morbidities | Framingham |
| Date of Exam 2 | HXMI | History of MI(REGARDS model) | Co-morbidities | Framingham |
| Date of Exam 3 | HXMI | History of MI(REGARDS model) | Co-morbidities | Framingham |
| Date of Exam 4 | HXMI | History of MI(REGARDS model) | Co-morbidities | Framingham |
| Date of Exam 5 | HXMI | History of MI(REGARDS model) | Co-morbidities | Framingham |
| Date of Exam 6 | HXMI | History of MI(REGARDS model) | Co-morbidities | Framingham |
| Date of Exam 7 | HXMI | History of MI(REGARDS model) | Co-morbidities | Framingham |
| Date of Exam 8 | HXMI | History of MI(REGARDS model) | Co-morbidities | Framingham |
| Date of Exam 9 | HXMI | History of MI(REGARDS model) | Co-morbidities | Framingham |
| Myocardial Infarction (MI) | HXMI | History of MI(REGARDS model) | Co-morbidities | MESA |
| Time to MI or End of Year 2015 (days) | HXMI | History of MI(REGARDS model) | Co-morbidities | MESA |
| Time between exam visits, in days | HXMI | History of MI(REGARDS model) | Co-morbidities | MESA |
| Time between first and third visits, in days | HXMI | History of MI(REGARDS model) | Co-morbidities | MESA |
| Time between first and fourth visits, in days | HXMI | History of MI(REGARDS model) | Co-morbidities | MESA |
| TIME BETWEEN FIRST AND FIFTH VISITS, IN DAYS | HXMI | History of MI(REGARDS model) | Co-morbidities | MESA |
| Hypertension by JNC VI (1997) criteria | HYPT | Hypertension (0=No, 1=Yes) | Co-morbidities | MESA |
| Hypertension by JNC VI (1997) criteria, Exam 2 | HYPT | Hypertension (0=No, 1=Yes) | Co-morbidities | MESA |
| Hypertension by JNC VI (1997) criteria, Exam 3 | HYPT | Hypertension (0=No, 1=Yes) | Co-morbidities | MESA |
| Hypertension by JNC VI (1997) criteria, Exam 4 | HYPT | Hypertension (0=No, 1=Yes) | Co-morbidities | MESA |
| HYPERTENSION BY JNC VI (1997) CRITERIA, EXAM 5 | HYPT | Hypertension (0=No, 1=Yes) | Co-morbidities | MESA |
| Scrambled Cohort Visit ID | ID_C | ARIC subject ID | Other | ARIC |
| MEDICATION & CONCENTRATION Q4M01A | INSULIN | Taking insulin (0=No, 1=Yes) | Medications | ARIC |
| MEDICATION & CONCENTRATION Q4M02A | INSULIN | Taking insulin (0=No, 1=Yes) | Medications | ARIC |
| MEDICATION & CONCENTRATION Q4M03A | INSULIN | Taking insulin (0=No, 1=Yes) | Medications | ARIC |
| MEDICATION & CONCENTRATION Q4M04A | INSULIN | Taking insulin (0=No, 1=Yes) | Medications | ARIC |
| MEDICATION & CONCENTRATION Q4M05A | INSULIN | Taking insulin (0=No, 1=Yes) | Medications | ARIC |
| MEDICATION & CONCENTRATION Q4M06A | INSULIN | Taking insulin (0=No, 1=Yes) | Medications | ARIC |
| MEDICATION & CONCENTRATION Q4M07A | INSULIN | Taking insulin (0=No, 1=Yes) | Medications | ARIC |
| MEDICATION & CONCENTRATION Q4M08A | INSULIN | Taking insulin (0=No, 1=Yes) | Medications | ARIC |
| MEDICATION & CONCENTRATION Q4M09A | INSULIN | Taking insulin (0=No, 1=Yes) | Medications | ARIC |
| MEDICATION & CONCENTRATION Q4M10A | INSULIN | Taking insulin (0=No, 1=Yes) | Medications | ARIC |
| MEDICATION & CONCENTRATION Q4M11A | INSULIN | Taking insulin (0=No, 1=Yes) | Medications | ARIC |
| MEDICATION & CONCENTRATION Q4M12A | INSULIN | Taking insulin (0=No, 1=Yes) | Medications | ARIC |
| MEDICATION & CONCENTRATION Q4M13A | INSULIN | Taking insulin (0=No, 1=Yes) | Medications | ARIC |
| MEDICATION & CONCENTRATION Q4M14A | INSULIN | Taking insulin (0=No, 1=Yes) | Medications | ARIC |
| MEDICATION & CONCENTRATION Q4M15A | INSULIN | Taking insulin (0=No, 1=Yes) | Medications | ARIC |
| MEDICATION & CONCENTRATION Q4M16A | INSULIN | Taking insulin (0=No, 1=Yes) | Medications | ARIC |
| MEDICATION & CONCENTRATION Q4M17A | INSULIN | Taking insulin (0=No, 1=Yes) | Medications | ARIC |
| MEDICATION NAME & CONCENTRATION Q4A | INSULIN | Taking insulin (0=No, 1=Yes) | Medications | ARIC |
| MEDICATION NAME & CONCENTRATION Q5A | INSULIN | Taking insulin (0=No, 1=Yes) | Medications | ARIC |
| MEDICATION NAME & CONCENTRATION Q6A | INSULIN | Taking insulin (0=No, 1=Yes) | Medications | ARIC |
| MEDICATION NAME & CONCENTRATION Q7A | INSULIN | Taking insulin (0=No, 1=Yes) | Medications | ARIC |
| MEDICATION NAME & CONCENTRATION Q8A | INSULIN | Taking insulin (0=No, 1=Yes) | Medications | ARIC |
| MEDICATION NAME & CONCENTRATION Q9A | INSULIN | Taking insulin (0=No, 1=Yes) | Medications | ARIC |
| MEDICATION NAME & CONCENTRATION Q10A | INSULIN | Taking insulin (0=No, 1=Yes) | Medications | ARIC |
| MEDICATION NAME & CONCENTRATION Q11A | INSULIN | Taking insulin (0=No, 1=Yes) | Medications | ARIC |
| MEDICATION NAME & CONCENTRATION Q12A | INSULIN | Taking insulin (0=No, 1=Yes) | Medications | ARIC |
| MEDICATION NAME & CONCENTRATION Q13A | INSULIN | Taking insulin (0=No, 1=Yes) | Medications | ARIC |
| MEDICATION NAME & CONCENTRATION Q14A | INSULIN | Taking insulin (0=No, 1=Yes) | Medications | ARIC |
| MEDICATION NAME & CONCENTRATION Q15A | INSULIN | Taking insulin (0=No, 1=Yes) | Medications | ARIC |
| MEDICATION NAME & CONCENTRATION Q16A | INSULIN | Taking insulin (0=No, 1=Yes) | Medications | ARIC |
| MEDICATION NAME & CONCENTRATION Q17A | INSULIN | Taking insulin (0=No, 1=Yes) | Medications | ARIC |
| MEDICATION NAME & CONCENTRATION Q18A | INSULIN | Taking insulin (0=No, 1=Yes) | Medications | ARIC |
| MEDICATION NAME & CONCENTRATION Q19A | INSULIN | Taking insulin (0=No, 1=Yes) | Medications | ARIC |
| MEDICATION NAME & CONCENTRATION Q20A | INSULIN | Taking insulin (0=No, 1=Yes) | Medications | ARIC |
| MEDICATION A NAME & CONCENTATION Q4A | INSULIN | Taking insulin (0=No, 1=Yes) | Medications | ARIC |
| MEDICATION B NAME & CONCENTRATION Q5A | INSULIN | Taking insulin (0=No, 1=Yes) | Medications | ARIC |
| MEDICATION C NAME & CONCENTRATION Q6A | INSULIN | Taking insulin (0=No, 1=Yes) | Medications | ARIC |
| MEDICATION D NAME & CONCENTRATION Q7A | INSULIN | Taking insulin (0=No, 1=Yes) | Medications | ARIC |
| MEDICATION E NAME & CONCENTRATION Q8A | INSULIN | Taking insulin (0=No, 1=Yes) | Medications | ARIC |
| MEDICATION F NAME & CONCENTRATION Q9A | INSULIN | Taking insulin (0=No, 1=Yes) | Medications | ARIC |
| MEDICATION G NAME & CONCENTRATION Q10A | INSULIN | Taking insulin (0=No, 1=Yes) | Medications | ARIC |
| MEDICATION H NAME & CONCENTRATION Q11A | INSULIN | Taking insulin (0=No, 1=Yes) | Medications | ARIC |
| MEDICATION I NAME & CONCENTRATION Q12A | INSULIN | Taking insulin (0=No, 1=Yes) | Medications | ARIC |
| MEDICATION J NAME & CONCENTRATION Q13A | INSULIN | Taking insulin (0=No, 1=Yes) | Medications | ARIC |
| MEDICATION K NAME & CONCENTRATION Q14A | INSULIN | Taking insulin (0=No, 1=Yes) | Medications | ARIC |
| MEDICATION L NAME & CONCENTRATION Q15A | INSULIN | Taking insulin (0=No, 1=Yes) | Medications | ARIC |
| MEDICATION M NAME & CONCENTRATION Q16A | INSULIN | Taking insulin (0=No, 1=Yes) | Medications | ARIC |
| MEDICATION N NAME & CONCENTRATION Q17A | INSULIN | Taking insulin (0=No, 1=Yes) | Medications | ARIC |
| MEDICATION P NAME & CONCENTRATION Q19A | INSULIN | Taking insulin (0=No, 1=Yes) | Medications | ARIC |
| MEDICATION Q NAME & CONCENTRATION Q20A | INSULIN | Taking insulin (0=No, 1=Yes) | Medications | ARIC |
| MEDICATION A NAME Q4A | INSULIN | Taking insulin (0=No, 1=Yes) | Medications | ARIC |
| MEDICATION B NAME Q5A | INSULIN | Taking insulin (0=No, 1=Yes) | Medications | ARIC |
| MEDICATION C NAME Q6A | INSULIN | Taking insulin (0=No, 1=Yes) | Medications | ARIC |
| MEDICATION D NAME Q7A | INSULIN | Taking insulin (0=No, 1=Yes) | Medications | ARIC |
| MEDICATION E NAME Q8A | INSULIN | Taking insulin (0=No, 1=Yes) | Medications | ARIC |
| MEDICATION F NAME Q9A | INSULIN | Taking insulin (0=No, 1=Yes) | Medications | ARIC |
| MEDICATION G NAME Q10A | INSULIN | Taking insulin (0=No, 1=Yes) | Medications | ARIC |
| MEDICATION H NAME Q11A | INSULIN | Taking insulin (0=No, 1=Yes) | Medications | ARIC |
| MEDICATION I NAME Q12A | INSULIN | Taking insulin (0=No, 1=Yes) | Medications | ARIC |
| MEDICATION J NAME Q13A | INSULIN | Taking insulin (0=No, 1=Yes) | Medications | ARIC |
| MEDICATION K NAME Q14A | INSULIN | Taking insulin (0=No, 1=Yes) | Medications | ARIC |
| MEDICATION L NAME Q15A | INSULIN | Taking insulin (0=No, 1=Yes) | Medications | ARIC |
| MEDICATION M NAME Q16A | INSULIN | Taking insulin (0=No, 1=Yes) | Medications | ARIC |
| MEDICATION N NAME Q17A | INSULIN | Taking insulin (0=No, 1=Yes) | Medications | ARIC |
| MEDICATION O NAME Q18A | INSULIN | Taking insulin (0=No, 1=Yes) | Medications | ARIC |
| MEDICATION P NAME Q19A | INSULIN | Taking insulin (0=No, 1=Yes) | Medications | ARIC |
| MEDICATION Q NAME Q20A | INSULIN | Taking insulin (0=No, 1=Yes) | Medications | ARIC |
| Med 1: Scanned UPC med name | INSULIN | Taking insulin (0=No, 1=Yes) | Medications | ARIC |
| Med 2: Scanned UPC med name | INSULIN | Taking insulin (0=No, 1=Yes) | Medications | ARIC |
| Med 3: Scanned UPC med name | INSULIN | Taking insulin (0=No, 1=Yes) | Medications | ARIC |
| Med 4: Scanned UPC med name | INSULIN | Taking insulin (0=No, 1=Yes) | Medications | ARIC |
| Med 5: Scanned UPC med name | INSULIN | Taking insulin (0=No, 1=Yes) | Medications | ARIC |
| Med 6: Scanned UPC med name | INSULIN | Taking insulin (0=No, 1=Yes) | Medications | ARIC |
| Med 7: Scanned UPC med name | INSULIN | Taking insulin (0=No, 1=Yes) | Medications | ARIC |
| Med 8: Scanned UPC med name | INSULIN | Taking insulin (0=No, 1=Yes) | Medications | ARIC |
| Med 9: Scanned UPC med name | INSULIN | Taking insulin (0=No, 1=Yes) | Medications | ARIC |
| Med 10: Scanned UPC med name | INSULIN | Taking insulin (0=No, 1=Yes) | Medications | ARIC |
| Med 11: Scanned UPC med name | INSULIN | Taking insulin (0=No, 1=Yes) | Medications | ARIC |
| Med 12: Scanned UPC med name | INSULIN | Taking insulin (0=No, 1=Yes) | Medications | ARIC |
| Med 13: Scanned UPC med name | INSULIN | Taking insulin (0=No, 1=Yes) | Medications | ARIC |
| Med 14: Scanned UPC med name | INSULIN | Taking insulin (0=No, 1=Yes) | Medications | ARIC |
| Med 15: Scanned UPC med name | INSULIN | Taking insulin (0=No, 1=Yes) | Medications | ARIC |
| Med 16: Scanned UPC med name | INSULIN | Taking insulin (0=No, 1=Yes) | Medications | ARIC |
| Med 17: Scanned UPC med name | INSULIN | Taking insulin (0=No, 1=Yes) | Medications | ARIC |
| Med 18: Scanned UPC med name | INSULIN | Taking insulin (0=No, 1=Yes) | Medications | ARIC |
| Med 19: Scanned UPC med name | INSULIN | Taking insulin (0=No, 1=Yes) | Medications | ARIC |
| Med 20: Scanned UPC med name | INSULIN | Taking insulin (0=No, 1=Yes) | Medications | ARIC |
| Med 21: Scanned UPC med name | INSULIN | Taking insulin (0=No, 1=Yes) | Medications | ARIC |
| Med 22: Scanned UPC med name | INSULIN | Taking insulin (0=No, 1=Yes) | Medications | ARIC |
| Med 23: Scanned UPC med name | INSULIN | Taking insulin (0=No, 1=Yes) | Medications | ARIC |
| Med 24: Scanned UPC med name | INSULIN | Taking insulin (0=No, 1=Yes) | Medications | ARIC |
| Med 25: Scanned UPC med name | INSULIN | Taking insulin (0=No, 1=Yes) | Medications | ARIC |
| D202-23-INSULIN | INSULIN | Taking insulin (0=No, 1=Yes) | Medications | Framingham |
| INSULIN | INSULIN | Taking insulin (0=No, 1=Yes) | Medications | Framingham |
| MEDS - INSULIN | INSULIN | Taking insulin (0=No, 1=Yes) | Medications | Framingham |
| ATC CODE FOR MEDICATION OR FIRST DRUG IN COMPOUND | INSULIN | Taking insulin (0=No, 1=Yes) | Medications | Framingham |
| ATC CODE FOR SECOND DRUG IN COMPOUND | INSULIN | Taking insulin (0=No, 1=Yes) | Medications | Framingham |
| ATC CODE FOR THIRD DRUG IN COMPOUND | INSULIN | Taking insulin (0=No, 1=Yes) | Medications | Framingham |
| ATC CODE FOR FOURTH DRUG IN COMPOUND | INSULIN | Taking insulin (0=No, 1=Yes) | Medications | Framingham |
| ATC code for medication or first drug in compound | INSULIN | Taking insulin (0=No, 1=Yes) | Medications | Framingham |
| ATC code for medication or second drug in compound | INSULIN | Taking insulin (0=No, 1=Yes) | Medications | Framingham |
| ATC code for medication or third drug in compound | INSULIN | Taking insulin (0=No, 1=Yes) | Medications | Framingham |
| ATC code for medication or fourth drug in compound | INSULIN | Taking insulin (0=No, 1=Yes) | Medications | Framingham |
| Insulins | INSULIN | Taking insulin (0=No, 1=Yes) | Medications | MESA |
| INSULINS | INSULIN | Taking insulin (0=No, 1=Yes) | Medications | MESA |
| RE-CALIBRATED LDL CHOL. in mg/dl | LDL | LDL cholesterol | Labs | ARIC |
| V2 re-calculated LDL-C in mg/dL | LDL | LDL cholesterol | Labs | ARIC |
| Re-Calibrated LDL Cholesterol in mg/dL | LDL | LDL cholesterol | Labs | ARIC |
| V4 Recalculated LDL Cholesterol | LDL | LDL cholesterol | Labs | ARIC |
| LDLCC-Cholesterol calculated (mg/dL) | LDL | LDL cholesterol | Labs | ARIC |
| LDL CHOLESTEROL (mg/dl) | LDL | LDL cholesterol | Labs | MESA |
| LDL Cholesterol (mg/dl) | LDL | LDL cholesterol | Labs | MESA |
| LVH present by cornell definition | LVH | Left ventricular hypertrophy(0=No, 1=Yes) | Co-morbidities | ARIC |
| Definite left ventricular hypertrophy, Exam 1 | LVH | Left ventricular hypertrophy(0=No, 1=Yes) | Co-morbidities | Framingham |
| Definite left ventricular hypertrophy, Exam 2 | LVH | Left ventricular hypertrophy(0=No, 1=Yes) | Co-morbidities | Framingham |
| Definite left ventricular hypertrophy, Exam 3 | LVH | Left ventricular hypertrophy(0=No, 1=Yes) | Co-morbidities | Framingham |
| Definite left ventricular hypertrophy, Exam 4 | LVH | Left ventricular hypertrophy(0=No, 1=Yes) | Co-morbidities | Framingham |
| Definite left ventricular hypertrophy, Exam 5 | LVH | Left ventricular hypertrophy(0=No, 1=Yes) | Co-morbidities | Framingham |
| Definite left ventricular hypertrophy, Exam 6 | LVH | Left ventricular hypertrophy(0=No, 1=Yes) | Co-morbidities | Framingham |
| Definite left ventricular hypertrophy, Exam 7 | LVH | Left ventricular hypertrophy(0=No, 1=Yes) | Co-morbidities | Framingham |
| Definite left ventricular hypertrophy, Exam 8 | LVH | Left ventricular hypertrophy(0=No, 1=Yes) | Co-morbidities | Framingham |
| Definite left ventricular hypertrophy, Exam 9 | LVH | Left ventricular hypertrophy(0=No, 1=Yes) | Co-morbidities | Framingham |
| LEFT VENTRICULAR HYPERTROPHY BY NOVACODE | LVH | Left ventricular hypertrophy(0=No, 1=Yes) | Co-morbidities | MESA |
| ECG LEFT VENTRICULAR HYPERTROPHY BY NOVACODE | LVH | Left ventricular hypertrophy(0=No, 1=Yes) | Co-morbidities | MESA |
| MESA Participant Identification Number | MESAID | MESA Participant Identification Number | Other | MESA |
| MESA PARTICIPANT IDENTIFICATION NUMBER | MESAID | MESA Participant Identification Number | Other | MESA |
| ANTI-CHOLESTEROL AGENTS | NONSTATIN | Taking non-statin medication (0=No, 1=Yes) | Medications | Framingham |
| D202-016-ANTI-CHOLESTEROL | NONSTATIN | Taking non-statin medication (0=No, 1=Yes) | Medications | Framingham |
| ANTI CHOLESTEROL DRUGS | NONSTATIN | Taking non-statin medication (0=No, 1=Yes) | Medications | Framingham |
| ANTI CHOLESTEROL DRUGS-RESINS | NONSTATIN | Taking non-statin medication (0=No, 1=Yes) | Medications | Framingham |
| ANTI CHOLESTEROL DRUGS-NIACIN | NONSTATIN | Taking non-statin medication (0=No, 1=Yes) | Medications | Framingham |
| ANTI CHOLESTEROL DRUGS-FIBRATES | NONSTATIN | Taking non-statin medication (0=No, 1=Yes) | Medications | Framingham |
| ANTI CHOLESTEROL DRUGS-OTHER | NONSTATIN | Taking non-statin medication (0=No, 1=Yes) | Medications | Framingham |
| MEDS - ANTI-CHOL -RESINS | NONSTATIN | Taking non-statin medication (0=No, 1=Yes) | Medications | Framingham |
| MEDS - ANTI-CHOL -NIACIN/NICOTINIC ACID | NONSTATIN | Taking non-statin medication (0=No, 1=Yes) | Medications | Framingham |
| MEDS - ANTI-CHOL -FIBRATES | NONSTATIN | Taking non-statin medication (0=No, 1=Yes) | Medications | Framingham |
| MEDS - ANTI-CHOL -OTHER | NONSTATIN | Taking non-statin medication (0=No, 1=Yes) | Medications | Framingham |
| MEDS - ANTI-CHOL - RESINS | NONSTATIN | Taking non-statin medication (0=No, 1=Yes) | Medications | Framingham |
| MEDS - ANTI-CHOL - NIACIN/NICOTINIC ACID | NONSTATIN | Taking non-statin medication (0=No, 1=Yes) | Medications | Framingham |
| MEDS - ANTI-CHOL - FIBRATES | NONSTATIN | Taking non-statin medication (0=No, 1=Yes) | Medications | Framingham |
| MEDS - ANTI-CHOL - OTHER | NONSTATIN | Taking non-statin medication (0=No, 1=Yes) | Medications | Framingham |
| ATC CODE FOR MEDICATION OR FIRST DRUG IN COMPOUND | NONSTATIN | Taking non-statin medication (0=No, 1=Yes) | Medications | Framingham |
| ATC CODE FOR SECOND DRUG IN COMPOUND | NONSTATIN | Taking non-statin medication (0=No, 1=Yes) | Medications | Framingham |
| ATC CODE FOR THIRD DRUG IN COMPOUND | NONSTATIN | Taking non-statin medication (0=No, 1=Yes) | Medications | Framingham |
| ATC CODE FOR FOURTH DRUG IN COMPOUND | NONSTATIN | Taking non-statin medication (0=No, 1=Yes) | Medications | Framingham |
| ATC code for medication or first drug in compound | NONSTATIN | Taking non-statin medication (0=No, 1=Yes) | Medications | Framingham |
| ATC code for medication or second drug in compound | NONSTATIN | Taking non-statin medication (0=No, 1=Yes) | Medications | Framingham |
| ATC code for medication or third drug in compound | NONSTATIN | Taking non-statin medication (0=No, 1=Yes) | Medications | Framingham |
| ATC code for medication or fourth drug in compound | NONSTATIN | Taking non-statin medication (0=No, 1=Yes) | Medications | Framingham |
| Bile-acid sequestrants | NONSTATIN | Taking non-statin medication (0=No, 1=Yes) | Medications | MESA |
| Fibrates | NONSTATIN | Taking non-statin medication (0=No, 1=Yes) | Medications | MESA |
| Miscellaneous lipid-lowering drugs | NONSTATIN | Taking non-statin medication (0=No, 1=Yes) | Medications | MESA |
| Niacin and nictotinic acid | NONSTATIN | Taking non-statin medication (0=No, 1=Yes) | Medications | MESA |
| Probucol | NONSTATIN | Taking non-statin medication (0=No, 1=Yes) | Medications | MESA |
| BILE-ACID SEQUESTRANTS | NONSTATIN | Taking non-statin medication (0=No, 1=Yes) | Medications | MESA |
| FIBRATES | NONSTATIN | Taking non-statin medication (0=No, 1=Yes) | Medications | MESA |
| MISCELLANEOUS LIPID-LOWERING DRUGS | NONSTATIN | Taking non-statin medication (0=No, 1=Yes) | Medications | MESA |
| NIACIN AND NICTOTINIC ACID | NONSTATIN | Taking non-statin medication (0=No, 1=Yes) | Medications | MESA |
| PROBUCOL | NONSTATIN | Taking non-statin medication (0=No, 1=Yes) | Medications | MESA |
| COHORT GENERAL EXAM DATA RANDOM ID | PID | UNIQUE PARTICIPANT ID | Other | Framingham |
| RACE (from FTRA23) | RACE_C | Race | Socio-demographics | ARIC |
| RACE | RACE_C | Race | Socio-demographics | MESA |
| SEX (from FTRA22) | SEX_C | Participant gender (character: F, M) | Socio-demographics | ARIC |
| Ã‚â€˜Participant genderÃ‚â€™ | SEX_C | Participant gender (character: F, M) | Socio-demographics | Framingham |
| gender | SEX_C | Participant gender (character: F, M) | Socio-demographics | MESA |
| SEX (from FTRA22) | SEX_N | Participant gender(0=Female, 1=Male) | Socio-demographics | ARIC |
| Ã‚â€˜Participant genderÃ‚â€™ | SEX_N | Participant gender(0=Female, 1=Male) | Socio-demographics | Framingham |
| gender | SEX_N | Participant gender(0=Female, 1=Male) | Socio-demographics | MESA |
| sodium (mg) | SODIUM | Sodium intake (mg/day) | Diet | ARIC |
| SODIUM (mg) | SODIUM | Sodium intake (mg/day) | Diet | Framingham |
| Sodium mg | SODIUM | Sodium intake (mg/day) | Diet | Framingham |
| Description not found | STATE | Site location (state) | Socio-demographics | ARIC |
| Exam 1: Site | STATE | Site location (state) | Socio-demographics | MESA |
| Used Statin (at Visit 1) last 2weeks (0=No, 1=Yes) based on 2004 Med Code | STATIN | Taking statin (0=No, 1=Yes) | Medications | ARIC |
| Used Statin (at Visit 2) last 2weeks (0=No, 1=Yes) based on 2004 Med Code | STATIN | Taking statin (0=No, 1=Yes) | Medications | ARIC |
| Used Statin (at Visit 3) last 2weeks (0=No, 1=Yes) based on 2004 Med Code | STATIN | Taking statin (0=No, 1=Yes) | Medications | ARIC |
| Used Statin (at Visit 4) last 2weeks (0=No, 1=Yes) based on 2004 Med Code | STATIN | Taking statin (0=No, 1=Yes) | Medications | ARIC |
| Med 1: Therapeutic Class Code | STATIN | Taking statin (0=No, 1=Yes) | Medications | ARIC |
| Med 2: Therapeutic Class Code | STATIN | Taking statin (0=No, 1=Yes) | Medications | ARIC |
| Med 3: Therapeutic Class Code | STATIN | Taking statin (0=No, 1=Yes) | Medications | ARIC |
| Med 4: Therapeutic Class Code | STATIN | Taking statin (0=No, 1=Yes) | Medications | ARIC |
| Med 5: Therapeutic Class Code | STATIN | Taking statin (0=No, 1=Yes) | Medications | ARIC |
| Med 6: Therapeutic Class Code | STATIN | Taking statin (0=No, 1=Yes) | Medications | ARIC |
| Med 7: Therapeutic Class Code | STATIN | Taking statin (0=No, 1=Yes) | Medications | ARIC |
| Med 8: Therapeutic Class Code | STATIN | Taking statin (0=No, 1=Yes) | Medications | ARIC |
| Med 9: Therapeutic Class Code | STATIN | Taking statin (0=No, 1=Yes) | Medications | ARIC |
| Med 10: Therapeutic Class Code | STATIN | Taking statin (0=No, 1=Yes) | Medications | ARIC |
| Med 11: Therapeutic Class Code | STATIN | Taking statin (0=No, 1=Yes) | Medications | ARIC |
| Med 12: Therapeutic Class Code | STATIN | Taking statin (0=No, 1=Yes) | Medications | ARIC |
| Med 13: Therapeutic Class Code | STATIN | Taking statin (0=No, 1=Yes) | Medications | ARIC |
| Med 14: Therapeutic Class Code | STATIN | Taking statin (0=No, 1=Yes) | Medications | ARIC |
| Med 15: Therapeutic Class Code | STATIN | Taking statin (0=No, 1=Yes) | Medications | ARIC |
| Med 16: Therapeutic Class Code | STATIN | Taking statin (0=No, 1=Yes) | Medications | ARIC |
| Med 17: Therapeutic Class Code | STATIN | Taking statin (0=No, 1=Yes) | Medications | ARIC |
| Med 18: Therapeutic Class Code | STATIN | Taking statin (0=No, 1=Yes) | Medications | ARIC |
| Med 19: Therapeutic Class Code | STATIN | Taking statin (0=No, 1=Yes) | Medications | ARIC |
| Med 20: Therapeutic Class Code | STATIN | Taking statin (0=No, 1=Yes) | Medications | ARIC |
| Med 21: Therapeutic Class Code | STATIN | Taking statin (0=No, 1=Yes) | Medications | ARIC |
| Med 22: Therapeutic Class Code | STATIN | Taking statin (0=No, 1=Yes) | Medications | ARIC |
| Med 23: Therapeutic Class Code | STATIN | Taking statin (0=No, 1=Yes) | Medications | ARIC |
| Med 24: Therapeutic Class Code | STATIN | Taking statin (0=No, 1=Yes) | Medications | ARIC |
| Med 25: Therapeutic Class Code | STATIN | Taking statin (0=No, 1=Yes) | Medications | ARIC |
| ANTI CHOLESTEROL DRUGS-STATINS | STATIN | Taking statin (0=No, 1=Yes) | Medications | Framingham |
| MEDS - ANTI-CHOL -STATINS | STATIN | Taking statin (0=No, 1=Yes) | Medications | Framingham |
| MEDS - ANTI-CHOL - STATINS | STATIN | Taking statin (0=No, 1=Yes) | Medications | Framingham |
| ATC CODE FOR MEDICATION OR FIRST DRUG IN COMPOUND | STATIN | Taking statin (0=No, 1=Yes) | Medications | Framingham |
| ATC CODE FOR SECOND DRUG IN COMPOUND | STATIN | Taking statin (0=No, 1=Yes) | Medications | Framingham |
| ATC CODE FOR THIRD DRUG IN COMPOUND | STATIN | Taking statin (0=No, 1=Yes) | Medications | Framingham |
| ATC CODE FOR FOURTH DRUG IN COMPOUND | STATIN | Taking statin (0=No, 1=Yes) | Medications | Framingham |
| ATC code for medication or first drug in compound | STATIN | Taking statin (0=No, 1=Yes) | Medications | Framingham |
| ATC code for medication or second drug in compound | STATIN | Taking statin (0=No, 1=Yes) | Medications | Framingham |
| ATC code for medication or third drug in compound | STATIN | Taking statin (0=No, 1=Yes) | Medications | Framingham |
| ATC code for medication or fourth drug in compound | STATIN | Taking statin (0=No, 1=Yes) | Medications | Framingham |
| HMG CoA reducatace inhibitors (statins) | STATIN | Taking statin (0=No, 1=Yes) | Medications | MESA |
| HMG COA REDUCATACE INHIBITORS (STATINS) | STATIN | Taking statin (0=No, 1=Yes) | Medications | MESA |
| ATC CODE FOR MEDICATION OR FIRST DRUG IN COMPOUND | STATNONSTAT | Taking statin/non-statin combination (0=No, 1=Yes) | Medications | Framingham |
| ATC CODE FOR SECOND DRUG IN COMPOUND | STATNONSTAT | Taking statin/non-statin combination (0=No, 1=Yes) | Medications | Framingham |
| ATC code for medication or first drug in compound | STATNONSTAT | Taking statin/non-statin combination (0=No, 1=Yes) | Medications | Framingham |
| ATC code for medication or second drug in compound | STATNONSTAT | Taking statin/non-statin combination (0=No, 1=Yes) | Medications | Framingham |
| 2ND AND 3RD SYSTOLIC BP AVERAGE Q21 | SYSBP | Seated systolic blood pressure (MM HG) | Vitals | ARIC |
| 2ND AND 3RD SYSTOLIC BP AVERAGE Q22 | SYSBP | Seated systolic blood pressure (MM HG) | Vitals | ARIC |
| AVG: Systolic (mmHg) | SYSBP | Seated systolic blood pressure (MM HG) | Vitals | ARIC |
| SEATED SYSTOLIC BLOOD PRESSURE (mmHg) | SYSBP | Seated systolic blood pressure (MM HG) | Vitals | MESA |
| Seated Systolic Blood Pressure (mmHg) | SYSBP | Seated systolic blood pressure (MM HG) | Vitals | MESA |
| Seated systolic blood pressure (mmhg) | SYSBP | Seated systolic blood pressure (MM HG) | Vitals | MESA |
| PHYSICIAN SYSTOLIC BLOOD PRESSURE, 1ST | SYSBP1 | PHYSICIAN SYSTOLIC BLOOD PRESSURE, FIRST (MM HG) | Vitals | Framingham |
| D201-047-SYS-PHYS-FIRST | SYSBP1 | PHYSICIAN SYSTOLIC BLOOD PRESSURE, FIRST (MM HG) | Vitals | Framingham |
| FIRST SYSTOLIC BLOOD PRESSURE | SYSBP1 | PHYSICIAN SYSTOLIC BLOOD PRESSURE, FIRST (MM HG) | Vitals | Framingham |
| SYSTOLIC BP - PHYSICIAN - 1ST READING | SYSBP1 | PHYSICIAN SYSTOLIC BLOOD PRESSURE, FIRST (MM HG) | Vitals | Framingham |
| SYSTOLIC PRESSURE - 1ST MD READING | SYSBP1 | PHYSICIAN SYSTOLIC BLOOD PRESSURE, FIRST (MM HG) | Vitals | Framingham |
| PHYSICIAN BP-SYSTOLIC-1ST READING | SYSBP1 | PHYSICIAN SYSTOLIC BLOOD PRESSURE, FIRST (MM HG) | Vitals | Framingham |
| PHYSICIAN BP SYSTOLIC - 1ST READING | SYSBP1 | PHYSICIAN SYSTOLIC BLOOD PRESSURE, FIRST (MM HG) | Vitals | Framingham |
| PHYSICIAN SYSTOLIC BP - 1ST READING | SYSBP1 | PHYSICIAN SYSTOLIC BLOOD PRESSURE, FIRST (MM HG) | Vitals | Framingham |
| MDBP1 - SYSTOLIC1, MMHG | SYSBP1 | PHYSICIAN SYSTOLIC BLOOD PRESSURE, FIRST (MM HG) | Vitals | Framingham |
| PHYSICIAN SYSTOLIC BLOOD PRESSURE, 2ND | SYSBP2 | PHYSICIAN SYSTOLIC BLOOD PRESSURE, SECOND (MM HG) | Vitals | Framingham |
| D201-053-SYS-PHYS-SECOND | SYSBP2 | PHYSICIAN SYSTOLIC BLOOD PRESSURE, SECOND (MM HG) | Vitals | Framingham |
| SECOND SYSTOLIC BLOOD PRESSURE | SYSBP2 | PHYSICIAN SYSTOLIC BLOOD PRESSURE, SECOND (MM HG) | Vitals | Framingham |
| SYSTOLIC BP - PHYSICIAN - 2ND READING | SYSBP2 | PHYSICIAN SYSTOLIC BLOOD PRESSURE, SECOND (MM HG) | Vitals | Framingham |
| SYSTOLIC PRESSURE - 2ND MD READING | SYSBP2 | PHYSICIAN SYSTOLIC BLOOD PRESSURE, SECOND (MM HG) | Vitals | Framingham |
| PHYSICIAN BP-2ND READING-SYSTOLIC | SYSBP2 | PHYSICIAN SYSTOLIC BLOOD PRESSURE, SECOND (MM HG) | Vitals | Framingham |
| PHYSICIAN BP SYSTOLIC - 2ND READING | SYSBP2 | PHYSICIAN SYSTOLIC BLOOD PRESSURE, SECOND (MM HG) | Vitals | Framingham |
| PHYSICIAN SYSTOLIC BP - 2ND READING | SYSBP2 | PHYSICIAN SYSTOLIC BLOOD PRESSURE, SECOND (MM HG) | Vitals | Framingham |
| MDBP2 - SYST, MMHG | SYSBP2 | PHYSICIAN SYSTOLIC BLOOD PRESSURE, SECOND (MM HG) | Vitals | Framingham |
| TOTAL CHOLESTEROL (MG-DL) | TC | Total cholesterol (mg/dL) | Labs | ARIC |
| CHOLESTEROL (VALUE) Q1A | TC | Total cholesterol (mg/dL) | Labs | ARIC |
| CHOLESTEROL (VALUE IN MG/DL) Q1A | TC | Total cholesterol (mg/dL) | Labs | ARIC |
| Total Cholesterol (mg/dL) | TC | Total cholesterol (mg/dL) | Labs | ARIC |
| Total cholesterol (mg/dL), Exam 1 | TC | Total cholesterol (mg/dL) | Labs | Framingham |
| Total cholesterol (mg/dL), Exam 2 | TC | Total cholesterol (mg/dL) | Labs | Framingham |
| Total cholesterol (mg/dL), Exam 3 | TC | Total cholesterol (mg/dL) | Labs | Framingham |
| Total cholesterol (mg/dL), Exam 4 | TC | Total cholesterol (mg/dL) | Labs | Framingham |
| Total cholesterol (mg/dL), Exam 5 | TC | Total cholesterol (mg/dL) | Labs | Framingham |
| Total cholesterol (mg/dL), Exam 6 | TC | Total cholesterol (mg/dL) | Labs | Framingham |
| Total cholesterol (mg/dL), Exam 7 | TC | Total cholesterol (mg/dL) | Labs | Framingham |
| Total cholesterol (mg/dL), Exam 8 | TC | Total cholesterol (mg/dL) | Labs | Framingham |
| Total cholesterol (mg/dL), Exam 9 | TC | Total cholesterol (mg/dL) | Labs | Framingham |
| TOTAL CHOLESTEROL (mg/dl) | TC | Total cholesterol (mg/dL) | Labs | MESA |
| Total Cholesterol (mg/dl) | TC | Total cholesterol (mg/dL) | Labs | MESA |
| TOTAL TRIGLYCERIDES (MG-DL) | TRIG | Total Triglycerides (mg/dL) | Labs | ARIC |
| TRIGLYCERIDE (VALUE) Q2A | TRIG | Total Triglycerides (mg/dL) | Labs | ARIC |
| TRIGLYCERIDE (VALUE IN MG/DL) Q2A | TRIG | Total Triglycerides (mg/dL) | Labs | ARIC |
| Total Triglycerides (mg/dL) | TRIG | Total Triglycerides (mg/dL) | Labs | ARIC |
| Triglycerides (mg/dL), Exam 1 | TRIG | Total Triglycerides (mg/dL) | Labs | Framingham |
| Triglycerides (mg/dL), Exam 2 | TRIG | Total Triglycerides (mg/dL) | Labs | Framingham |
| Triglycerides (mg/dL), Exam 3 | TRIG | Total Triglycerides (mg/dL) | Labs | Framingham |
| Triglycerides (mg/dL), Exam 4 | TRIG | Total Triglycerides (mg/dL) | Labs | Framingham |
| Triglycerides (mg/dL), Exam 5 | TRIG | Total Triglycerides (mg/dL) | Labs | Framingham |
| Triglycerides (mg/dL), Exam 6 | TRIG | Total Triglycerides (mg/dL) | Labs | Framingham |
| Triglycerides (mg/dL), Exam 7 | TRIG | Total Triglycerides (mg/dL) | Labs | Framingham |
| Triglycerides (mg/dL), Exam 8 | TRIG | Total Triglycerides (mg/dL) | Labs | Framingham |
| Triglycerides (mg/dL), Exam 9 | TRIG | Total Triglycerides (mg/dL) | Labs | Framingham |
| TRIGLYCERIDES (mg/dl) | TRIG | Total Triglycerides (mg/dL) | Labs | MESA |
| Triglycerides (mg/dl) | TRIG | Total Triglycerides (mg/dL) | Labs | MESA |
| CDI: RHEUMATIC HEART DISEASE | VALVDIS | Valvular heart disease (0=No, 1=Yes) | Co-morbidities | Framingham |
| CDI: AORTIC VALVE DISEASE | VALVDIS | Valvular heart disease (0=No, 1=Yes) | Co-morbidities | Framingham |
| CDI: MITRAL VALVE DISEASE | VALVDIS | Valvular heart disease (0=No, 1=Yes) | Co-morbidities | Framingham |
| CDI-RHEUMATIC HEART DISEASE | VALVDIS | Valvular heart disease (0=No, 1=Yes) | Co-morbidities | Framingham |
| CDI-AORTIC VALVE DISEASE | VALVDIS | Valvular heart disease (0=No, 1=Yes) | Co-morbidities | Framingham |
| CDI-MITRAL VALVE DISEASE | VALVDIS | Valvular heart disease (0=No, 1=Yes) | Co-morbidities | Framingham |
| CDI - RHEUMATIC HEART DISEASE | VALVDIS | Valvular heart disease (0=No, 1=Yes) | Co-morbidities | Framingham |
| CDI - AORTIC VALVE DISEASE | VALVDIS | Valvular heart disease (0=No, 1=Yes) | Co-morbidities | Framingham |
| CDI - MITRAL VALVE DISEASE | VALVDIS | Valvular heart disease (0=No, 1=Yes) | Co-morbidities | Framingham |
| RHEUMATIC HEART DISEASE: SELF-REPORT | VALVDIS | Valvular heart disease (0=No, 1=Yes) | Co-morbidities | MESA |
| CONSUMED STRING OR GREEN BEANS Q15 | VEGETABLES | Vegetables (servings per week) | Diet | ARIC |
| CONSUMED BROCCOLI Q16 | VEGETABLES | Vegetables (servings per week) | Diet | ARIC |
| CABBAGE, CAULIFLWR,BUS SPROUT Q17 | VEGETABLES | Vegetables (servings per week) | Diet | ARIC |
| CONSUMED CARROTS Q18 | VEGETABLES | Vegetables (servings per week) | Diet | ARIC |
| CONSUMED CORN Q19 | VEGETABLES | Vegetables (servings per week) | Diet | ARIC |
| SPINACH, COLLARDS, GREENS Q20 | VEGETABLES | Vegetables (servings per week) | Diet | ARIC |
| CONSUMED PEAS OR LIMA BEANS Q21 | VEGETABLES | Vegetables (servings per week) | Diet | ARIC |
| CONSUMED DARK YELLOW SQUASH Q22 | VEGETABLES | Vegetables (servings per week) | Diet | ARIC |
| CONSUMED SWEET POTATOES Q23 | VEGETABLES | Vegetables (servings per week) | Diet | ARIC |
| CONSUMED BEANS OR LENTILS Q24 | VEGETABLES | Vegetables (servings per week) | Diet | ARIC |
| CONSUMED TOMATOES Q25 | VEGETABLES | Vegetables (servings per week) | Diet | ARIC |
| CONSUMED STRING OR GREEN BEANS Q15 | VEGETABLES | Vegetables (servings per week) | Diet | ARIC |
| CONSUMED BROCCOLI Q16 | VEGETABLES | Vegetables (servings per week) | Diet | ARIC |
| CABBAGE, CAULIFLWR, BRUS SPROUTS Q17 | VEGETABLES | Vegetables (servings per week) | Diet | ARIC |
| CONSUMED CARROTS Q18 | VEGETABLES | Vegetables (servings per week) | Diet | ARIC |
| CONSUMED CORN Q19 | VEGETABLES | Vegetables (servings per week) | Diet | ARIC |
| SPINACH, COLLARDS, GREENS Q20 | VEGETABLES | Vegetables (servings per week) | Diet | ARIC |
| CONSUMED PEAS OR LIMA BEANS Q21 | VEGETABLES | Vegetables (servings per week) | Diet | ARIC |
| CONSUMED DARK YELLOW SQUASH Q22 | VEGETABLES | Vegetables (servings per week) | Diet | ARIC |
| CONSUMED SWEET POTATOES Q23 | VEGETABLES | Vegetables (servings per week) | Diet | ARIC |
| CONSUMED BEANS OR LENTILS Q24 | VEGETABLES | Vegetables (servings per week) | Diet | ARIC |
| CONSUMED TOMATOES Q25 | VEGETABLES | Vegetables (servings per week) | Diet | ARIC |
| GREEN STRING BEANS OR ASPARAGUS | VEGETABLES | Vegetables (servings per week) | Diet | Framingham |
| HALF CUP BROCCOLI | VEGETABLES | Vegetables (servings per week) | Diet | Framingham |
| CABBAGE COLE SLAW OR SAUERKRAUT | VEGETABLES | Vegetables (servings per week) | Diet | Framingham |
| CAULIFLOWER-HALF CUP | VEGETABLES | Vegetables (servings per week) | Diet | Framingham |
| HALF CUP BRUSSEL SPROUTS | VEGETABLES | Vegetables (servings per week) | Diet | Framingham |
| ONE WHOLE OR HALF CUP COOKED | VEGETABLES | Vegetables (servings per week) | Diet | Framingham |
| EAR OR HALF CUP FROZEN | VEGETABLES | Vegetables (servings per week) | Diet | Framingham |
| HALF CUP SPINACH RAW OR COOKED | VEGETABLES | Vegetables (servings per week) | Diet | Framingham |
| GREEN OR RED PEPPERS-HALF CUP | VEGETABLES | Vegetables (servings per week) | Diet | Framingham |
| KALE,MUSTARD,CHARD,BEET OR OTHER GREENS | VEGETABLES | Vegetables (servings per week) | Diet | Framingham |
| CUP OF ICEBERG OR HEAD LETTUCE | VEGETABLES | Vegetables (servings per week) | Diet | Framingham |
| ESCAROLE,ROMAINE,WATERCRESS LEAFLETTUCE | VEGETABLES | Vegetables (servings per week) | Diet | Framingham |
| PEAS,LIMA BEANS OR PEA PODS | VEGETABLES | Vegetables (servings per week) | Diet | Framingham |
| YELLOW WINTER SQUASH OR PUMPKINS | VEGETABLES | Vegetables (servings per week) | Diet | Framingham |
| EGGPLANT,ZUCCHINI,OTHER SUMMER SQUASH | VEGETABLES | Vegetables (servings per week) | Diet | Framingham |
| HALF CUP YAMS OR SWEET POTATOES | VEGETABLES | Vegetables (servings per week) | Diet | Framingham |
| ONE TOMATO OR HALF CUP | VEGETABLES | Vegetables (servings per week) | Diet | Framingham |
| LENTILS, CHICK PEAS, KIDNEY,PINTO BEANS | VEGETABLES | Vegetables (servings per week) | Diet | Framingham |
| TOMATOES:(servings/week) | VEGETABLES | Vegetables (servings per week) | Diet | Framingham |
| STRING BEANS:(servings/week) | VEGETABLES | Vegetables (servings per week) | Diet | Framingham |
| BROCCOLI:(servings/week) | VEGETABLES | Vegetables (servings per week) | Diet | Framingham |
| CABBAGE/COLE SLAW:(servings/week) | VEGETABLES | Vegetables (servings per week) | Diet | Framingham |
| CAULIFLOWER:(servings/week) | VEGETABLES | Vegetables (servings per week) | Diet | Framingham |
| BRUSSELS SPROUTS:(servings/week) | VEGETABLES | Vegetables (servings per week) | Diet | Framingham |
| CARROTS, RAW:(servings/week) | VEGETABLES | Vegetables (servings per week) | Diet | Framingham |
| CARROTS, COOKED:(servings/week) | VEGETABLES | Vegetables (servings per week) | Diet | Framingham |
| CORN:(servings/week) | VEGETABLES | Vegetables (servings per week) | Diet | Framingham |
| PEAS/LIMA BEANS:(servings/week) | VEGETABLES | Vegetables (servings per week) | Diet | Framingham |
| MIXED VEGETABLES:(servings/week) | VEGETABLES | Vegetables (servings per week) | Diet | Framingham |
| BEANS/LENTILS:(servings/week) | VEGETABLES | Vegetables (servings per week) | Diet | Framingham |
| WINTER SQUASH:(servings/week) | VEGETABLES | Vegetables (servings per week) | Diet | Framingham |
| SUMMER SQUASH:(servings/week) | VEGETABLES | Vegetables (servings per week) | Diet | Framingham |
| YAMS/SWEET POTATOES:(servings/week) | VEGETABLES | Vegetables (servings per week) | Diet | Framingham |
| SPINACH, COOKED:(servings/week) | VEGETABLES | Vegetables (servings per week) | Diet | Framingham |
| SPANACH, RAW:(servings/week) | VEGETABLES | Vegetables (servings per week) | Diet | Framingham |
| KALE/MUSTARD/CHARD:(servings/week) | VEGETABLES | Vegetables (servings per week) | Diet | Framingham |
| ICEBERG/HEAD LETTUCE:(servings/week) | VEGETABLES | Vegetables (servings per week) | Diet | Framingham |
| ROMAINE/LEAF LETTUCE:(servings/week) | VEGETABLES | Vegetables (servings per week) | Diet | Framingham |
| CELERY:(servings/week) | VEGETABLES | Vegetables (servings per week) | Diet | Framingham |
| BEETS:(servings/week) | VEGETABLES | Vegetables (servings per week) | Diet | Framingham |
| DAYS SINCE EXAM 1 | VISDAY | Visit day (days since exam 1) | Other | ARIC |
| Visit 5 Date, NCS Stage 1 - days from visit 1 | VISDAY | Visit day (days since exam 1) | Other | ARIC |
| Date of Exam 2 | VISDAY | Visit day (days since exam 1) | Other | Framingham |
| Date of Exam 3 | VISDAY | Visit day (days since exam 1) | Other | Framingham |
| Date of Exam 4 | VISDAY | Visit day (days since exam 1) | Other | Framingham |
| Date of Exam 5 | VISDAY | Visit day (days since exam 1) | Other | Framingham |
| Date of Exam 6 | VISDAY | Visit day (days since exam 1) | Other | Framingham |
| Date of Exam 7 | VISDAY | Visit day (days since exam 1) | Other | Framingham |
| Date of Exam 8 | VISDAY | Visit day (days since exam 1) | Other | Framingham |
| Date of Exam 9 | VISDAY | Visit day (days since exam 1) | Other | Framingham |
| Time between exam visits, in days | VISDAY | Visit day (days since exam 1) | Other | MESA |
| Time between first and third visits, in days | VISDAY | Visit day (days since exam 1) | Other | MESA |
| Time between first and fourth visits, in days | VISDAY | Visit day (days since exam 1) | Other | MESA |
| TIME BETWEEN FIRST AND FIFTH VISITS, IN DAYS | VISDAY | Visit day (days since exam 1) | Other | MESA |
| WEIGHT TO THE NEAREST LB Q4 | WGT | Weight (to the nearest lb) | Vitals | ARIC |
| WEIGHT TO NEAREST LB Q1 | WGT | Weight (to the nearest lb) | Vitals | ARIC |
| WEIGHT TO NEAREST LB Q2 | WGT | Weight (to the nearest lb) | Vitals | ARIC |
| Weight (kg) | WGT | Weight (to the nearest lb) | Vitals | ARIC |
| Weight (pounds), Exam 1 | WGT | Weight (to the nearest lb) | Vitals | Framingham |
| Weight (pounds), Exam 2 | WGT | Weight (to the nearest lb) | Vitals | Framingham |
| Weight (pounds), Exam 3 | WGT | Weight (to the nearest lb) | Vitals | Framingham |
| Weight (pounds), Exam 4 | WGT | Weight (to the nearest lb) | Vitals | Framingham |
| Weight (pounds), Exam 5 | WGT | Weight (to the nearest lb) | Vitals | Framingham |
| Weight (pounds), Exam 6 | WGT | Weight (to the nearest lb) | Vitals | Framingham |
| Weight (pounds), Exam 7 | WGT | Weight (to the nearest lb) | Vitals | Framingham |
| Weight (pounds), Exam 8 | WGT | Weight (to the nearest lb) | Vitals | Framingham |
| Weight (pounds), Exam 9 | WGT | Weight (to the nearest lb) | Vitals | Framingham |
| WEIGHT (lbs) | WGT | Weight (to the nearest lb) | Vitals | MESA |

**Supplemental Table 2**: Per-concept AUC for the trained models.

| **Concept Name** | **Baseline Model AUC** | **FCN Model (Base) AUC** | **FCN Model (Contrastive Learning) AUC** |
| --- | --- | --- | --- |
| AFIB | 0.5940 | 0.9647 | 0.9603 |
| AGE | 0.9677 | 1.0000 | 1.0000 |
| ALCOHOL | 0.8357 | 1.0000 | 0.9974 |
| ANYCHOLMED | 0.7396 | 0.9651 | 0.9422 |
| ASPIRIN | 0.6668 | 0.9203 | 0.9335 |
| BASE_CVD | 0.6944 | 0.9722 | 0.9333 |
| BASE_STROKE | 0.7063 | 1.0000 | 0.9781 |
| BG | 0.7795 | 1.0000 | 1.0000 |
| BMI | 0.7665 | 1.0000 | 0.9857 |
| CARSTEN | 0.6933 | 1.0000 | 1.0000 |
| CENSDAY | 0.9722 | 0.9792 | 1.0000 |
| CREAT | 0.8900 | 1.0000 | 1.0000 |
| CURRSMK | 0.9227 | 1.0000 | 1.0000 |
| DAYS_SINCE_EXAM1 | NA | NA | NA |
| DEATH_IND | 0.9387 | 1.0000 | 1.0000 |
| DEATH_IND_T2 | NA | NA | NA |
| DIAB | 0.8318 | 1.0000 | 0.9317 |
| DIABP | 0.7595 | 1.0000 | 1.0000 |
| DIABP1 | 0.9718 | 0.9993 | 0.9987 |
| DIABP2 | 0.8849 | 1.0000 | 0.9911 |
| EDUCLEV | 0.1489 | 1.0000 | 0.8085 |
| EVENT | NA | NA | NA |
| EVENT_DESC | 0.4444 | 0.8518 | 0.9259 |
| EVENT_T2_O | 0.9480 | 1.0000 | 0.9954 |
| EVENT_VAL_C | 0.6000 | 0.9938 | 0.9954 |
| FAM_INCOME | 0.5727 | 1.0000 | 1.0000 |
| FASTING_12HR | 0.3415 | 1.0000 | 1.0000 |
| FASTING_8HR | 1.000 | 1.0000 | 1.0000 |
| FASTING_BG | 0.9848 | 1.0000 | 1.0000 |
| FH_STROKE | 0.7024 | 0.9821 | 0.9940 |
| FRUITS | 0.8183 | 1.0000 | 0.9566 |
| GENHLTH | NA | NA | NA |
| GENHLTH2 | 0.5593 | 1.0000 | 1.0000 |
| GLUCOSE | 0.6988 | 1.0000 | 1.0000 |
| HDL | 0.9403 | 1.0000 | 1.0000 |
| HGT_CM | 0.6426 | 0.9917 | 0.9966 |
| HRX | 0.9295 | 1.0000 | 0.9996 |
| HXCVD | 0.6779 | 0.9380 | 0.9327 |
| HXHRTD | 0.6588 | 0.9545 | 0.9376 |
| HXMI | 0.6898 | 0.9484 | 0.9500 |
| HYPT | 0.5034 | 0.9899 | 1.0000 |
| ID_C | NA | NA | NA |
| INSULIN | 0.8899 | 0.9935 | 0.9917 |
| LDL | 0.8851 | 0.9836 | 0.9918 |
| LVH | 0.9394 | 1.0000 | 0.9963 |
| MESAID | 1.0000 | 1.0000 | 1.0000 |
| NONSTATIN | 0.6652 | 0.9465 | 0.9155 |
| PID | NA | NA | NA |
| RACE_C | NA | NA | NA |
| SEX_C | 0.6400 | 1.0000 | 1.0000 |
| SEX_N | NA | NA | NA |
| SODIUM | 1.0000 | 1.0000 | 1.0000 |
| STATE | 0.5135 | 1.0000 | 1.0000 |
| STATIN | 0.8710 | 0.9743 | 0.9826 |
| STATNONSTAT | 0.8257 | 0.8030 | 0.8485 |
| SYSBP | 0.6824 | 1.0000 | 0.9882 |
| SYSBP1 | 0.8361 | 1.0000 | 0.9958 |
| SYSBP2 | 0.8106 | 1.0000 | 0.9961 |
| TC | 0.8462 | 1.0000 | 0.9998 |
| TRIG | 0.9025 | 1.0000 | 1.0000 |
| VALVDIS | 0.9383 | 1.0000 | 0.9872 |
| VEGETABLES | 0.9033 | 1.0000 | 0.9846 |
| VISDAY | 0.8510 | 0.9210 | 0.9334 |
| WGT | 0.8038 | 0.9999 | 1.0000 |

* For the AUCs listed as NA, there were only single classes present in the test set, and therefore the AUCs are undefined.

**Supplemental Table 3:** Per-concept metrics for accuracy, precision, and recall using Top-1 concept prediction.

| **Concept** | **True Positive Rate (TPR)** | **True Negative Rate (TNR)** | **Positive Predictive Value (PPV)** | **Negative Predictive Value (NPV)** | **False Positive Rate (FPR)** | **False Negative Rate (FNR)** | **False Discovery Rate (FDR)** | **Accuracy (ACC)** |
| --- | --- | --- | --- | --- | --- | --- | --- | --- |
| AFIB | 1 | 0.9946 | 0.7368 | 1 | 0.0053 | 0 | 0.2631 | 0.9947 |
| AGE | 0.9523 | 1 | 1 | 0.9989 | 0 | 0.0476 | 0 | 0.9989 |
| ALCOHOL | 1 | 1 | 1 | 1 | 0 | 0 | 0 | 1 |
| ANYCHOLMED | 0.6521 | 0.9665 | 0.3260 | 0.9911 | 0.0334 | 0.3478 | 0.6739 | 0.9589 |
| ASPIRIN | 0.7727 | 0.9924 | 0.7083 | 0.9945 | 0.0075 | 0.2272 | 0.2916 | 0.9873 |
| BASE_CVD | 0.6666 | 0.9957 | 0.5 | 0.9978 | 0.0042 | 0.3333 | 0.5 | 0.9936 |
| BASE_STROKE | 1 | 0.9978 | 0.7142 | 1 | 0.0021 | 0 | 0.2857 | 0.9978 |
| BG | 1 | 1 | 1 | 1 | 0 | 0 | 0 | 1 |
| BMI | 0.8888 | 1 | 1 | 0.9978 | 0 | 0.1111 | 0 | 0.9978 |
| CARSTEN | 1 | 1 | 1 | 1 | 0 | 0 | 0 | 1 |
| CENSDAY | 0.375 | 0.9978 | 0.6 | 0.9947 | 0.0021 | 0.625 | 0.4 | 0.9926 |
| CREAT | 1 | 1 | 1 | 1 | 0 | 0 | 0 | 1 |
| CURRSMK | 1 | 1 | 1 | 1 | 0 | 0 | 0 | 1 |
| DAYS_SINCE_EXAM1 | 1 | 0.9989 | 0.5 | 1 | 0.001 | 0 | 0.5 | 0.9989 |
| DEATH_IND | 0.6666 | 0.9978 | 0.5 | 0.9989 | 0.0021 | 0.3333 | 0.5 | 0.9968 |
| DEATH_IND_T2 | 1 | 0.9989 | 0.5 | 1 | 0.001 | 0 | 0.5 | 0.9989 |
| DIAB | 1 | 1 | 1 | 1 | 0 | 0 | 0 | 1 |
| DIABP | 1 | 0.9968 | 0.625 | 1 | 0.0031 | 0 | 0.375 | 0.9968 |
| DIABP1 | 0.9090 | 1 | 1 | 0.9989 | 0 | 0.0909 | 0 | 0.9989 |
| DIABP2 | 0.9090 | 1 | 1 | 0.9989 | 0 | 0.0909 | 0 | 0.9989 |
| EDUCLEV | 1 | 1 | 1 | 1 | 0 | 0 | 0 | 1 |
| EVENT | 1 | 0.9989 | 0.5 | 1 | 0.001 | 0 | 0.5 | 0.9989 |
| EVENT_DESC | 0.5 | 0.9989 | 0.5 | 0.9989 | 0.001 | 0.5 | 0.5 | 0.9978 |
| EVENT_T2_O | 0.8888 | 0.9989 | 0.8888 | 0.9989 | 0.001 | 0.1111 | 0.1111 | 0.9978 |
| EVENT_VAL_C | 0.7777 | 0.9968 | 0.7 | 0.9978 | 0.0031 | 0.2222 | 0.3 | 0.9947 |
| FAM_INCOME | 0.6 | 0.9978 | 0.6 | 0.9978 | 0.0021 | 0.4 | 0.4 | 0.9957 |
| FASTING_12HR | 1 | 1 | 1 | 1 | 0 | 0 | 0 | 1 |
| FASTING_8HR | 1 | 1 | 1 | 1 | 0 | 0 | 0 | 1 |
| FASTING_BG | 0.75 | 1 | 1 | 0.9957 | 0 | 0.25 | 0 | 0.9957 |
| FH_STROKE | 0.8333 | 0.9989 | 0.8333 | 0.9989 | 0.001 | 0.1666 | 0.1667 | 0.9978 |
| FRUITS | 1 | 1 | 1 | 1 | 0 | 0 | 0 | 1 |
| GENHLTH | 0.5 | 1 | 1 | 0.9968 | 0 | 0.5 | 0 | 0.9968 |
| GENHLTH2 | 1 | 0.9989 | 0.8 | 1 | 0.001 | 0 | 0.2 | 0.9989 |
| GLUCOSE | 1 | 0.9957 | 0.3333 | 1 | 0.0042 | 0 | 0.6667 | 0.9957 |
| HDL | 1 | 1 | 1 | 1 | 0 | 0 | 0 | 1 |
| HGT_CM | 1 | 0.9989 | 0.9285 | 1 | 0.001 | 0 | 0.0714 | 0.9989 |
| HRX | 0.9473 | 1 | 1 | 0.9989 | 0 | 0.0526 | 0 | 0.9989 |
| HXCVD | 0.3571 | 0.9776 | 0.5 | 0.9603 | 0.0223 | 0.6428 | 0.5 | 0.9409 |
| HXHRTD | 0.4347 | 0.9701 | 0.4255 | 0.9711 | 0.0299 | 0.5652 | 0.5744 | 0.9441 |
| HXMI | 0.7368 | 0.9827 | 0.4666 | 0.9945 | 0.0172 | 0.2631 | 0.5333 | 0.9778 |
| HYPT | 0.8333 | 0.9989 | 0.8333 | 0.9989 | 0.001 | 0.1666 | 0.1667 | 0.9978 |
| ID_C | 1 | 1 | 1 | 1 | 0 | 0 | 0 | 1 |
| INSULIN | 1 | 0.9905 | 0.9245 | 1 | 0.0094 | 0 | 0.0754 | 0.9915 |
| LDL | 1 | 0.9978 | 0.75 | 1 | 0.0021 | 0 | 0.25 | 0.9978 |
| LVH | 1 | 1 | 1 | 1 | 0 | 0 | 0 | 1 |
| MESAID | 0.75 | 1 | 1 | 0.9989 | 0 | 0.25 | 0 | 0.9989 |
| NONSTATIN | 0.5 | 0.9824 | 0.5294 | 0.9803 | 0.0175 | 0.5 | 0.4705 | 0.9641 |
| PID | 1 | 0.9989 | 0.5 | 1 | 0.001 | 0 | 0.5 | 0.9989 |
| RACE_C | 1 | 0.9989 | 0.6666 | 1 | 0.001 | 0 | 0.3333 | 0.9989 |
| SEX_C | 0.5 | 0.9968 | 0.25 | 0.9989 | 0.0031 | 0.5 | 0.75 | 0.9957 |
| SEX_N | 0.5714 | 1 | 1 | 0.9968 | 0 | 0.4285 | 0 | 0.9968 |
| SODIUM | 1 | 1 | 1 | 1 | 0 | 0 | 0 | 1 |
| STATE | 1 | 0.997 | 0.3333 | 1 | 0.0021 | 0 | 0.6667 | 0.9978 |
| STATIN | 0.5384 | 0.9988 | 0.9767 | 0.9602 | 0.0011 | 0.4615 | 0.0232 | 0.961 |
| STATNONSTAT | 1 | 0.9957 | 0.2 | 1 | 0.0042 | 0 | 0.8 | 0.9957 |
| SYSBP | 1 | 1 | 1 | 1 | 0 | 0 | 0 | 1 |
| SYSBP1 | 1 | 1 | 1 | 1 | 0 | 0 | 0 | 1 |
| SYSBP2 | 1 | 1 | 1 | 1 | 0 | 0 | 0 | 1 |
| TC | 1 | 1 | 1 | 1 | 0 | 0 | 0 | 1 |
| TRIG | 1 | 1 | 1 | 1 | 0 | 0 | 0 | 1 |
| VALVDIS | 1 | 1 | 1 | 1 | 0 | 0 | 0 | 1 |
| VEGETABLES | 1 | 1 | 1 | 1 | 0 | 0 | 0 | 1 |
| VISDAY | 0.25 | 0.9892 | 0.3333 | 0.9839 | 0.0107 | 0.75 | 0.6667 | 0.9736 |
| WGT | 0.8823 | 1 | 1 | 0.9978 | 0 | 0.1176 | 0 | 0.9978 |
